# Expression of 1,3-β-glucan synthase subunits in *Candida glabrata* is regulated by the cell cycle and growth conditions and at both transcriptional and post-transcriptional levels

**DOI:** 10.1128/aac.00500-25

**Published:** 2025-06-17

**Authors:** Irene Gonzalez-Jimenez, Mikhail V. Keniya, Ariel A. Aptekmann, Christopher Quinteros, Alexis Wilkerson, Amir Arastehfar, Farnaz Daneshnia, David S. Perlin, Erika Shor

**Affiliations:** 1 Hackensack Meridian Health Center for Discovery and Innovation, Hackensack Meridian School of Medicine 576909 , , Nutley, New Jersey, USA; 2 Department of Medical Sciences, Hackensack Meridian School of Medicine 576909 , Nutley, New Jersey, USA; 3 Georgetown University Lombardi Comprehensive Cancer Center, Georgetown University Lombardi Comprehensive Cancer Center 66634, Washington, District of Columbia, USA; University Children's Hospital Münster, Münster, Germany

**Keywords:** regulation of expression, FKS1, FKS2, *Candida glabrata*, glucan synthase

## Abstract

Fungal cell wall-synthesizing enzyme 1,3-β-glucan synthase (GS) is the target of the echinocandins, a frontline antifungal drug class. However, increasing echinocandin resistance due to mutations in GS has been observed in certain fungal pathogens, notably *Candida glabrata*, where GS is encoded by two homologous genes, *FKS1* and *FKS2*. Despite the importance of GS in the fungal life cycle and as a drug target, the regulation of its expression in culture and in the host is still poorly understood. In this study, we used a fluorescent transcriptional reporter, quantitative reverse-transcriptase PCR, protein analysis, and mining of RNA-seq data sets to examine the regulation of *C. glabrata* GS expression. We determined that *FKS1* and *FKS2* promoter activities peak during S-phase and that during exponential growth in culture, *FKS1* is expressed at higher levels than *FKS2*. Interestingly, although *FKS*2 mRNA expression appeared to be strongly induced in an *fks1∆* mutant, this calcineurin-mediated induction was not accompanied by increased *FKS2* promoter activity, suggesting post-transcriptional regulation. Examination of *FKS2* transcript across the ORF as well as Fks2 protein levels was consistent with post-transcriptional regulation. Finally, RNA-seq data mining revealed that, in contrast to vegetative growth, during the stationary phase and under host conditions, *FKS2* is expressed at equivalent or higher levels than *FKS1*. Together, these experiments revealed that in *C. glabrata,* the expression of both GS subunits is regulated transcriptionally by the cell cycle, that *FKS2* expression is regulated post-transcriptionally by calcineurin and *fks1∆*, and that in the host, *FKS2* may be the more abundant subunit.

## INTRODUCTION

β-1,3-glucan synthase (GS) is a fungal enzyme essential for cell wall biosynthesis and the target of echinocandins, a frontline antifungal drug class. Given the increasing resistance to azole-class drugs among major fungal pathogens, coupled with their established safety and minimal drug-drug interactions, echinocandins are now the recommended first-line therapy for invasive fungal infections (1). However, increasing echinocandin resistance due to mutations in GS has been reported in a few species, most notably *Candida glabrata* (also referred to as *Nakaseomyces glabratus*) (2–10). In *C. glabrata* and in the closely related non-pathogenic yeast *Saccharomyces cerevisiae,* GS is encoded by two functionally redundant paralogous genes, *FKS1* (a.k.a. *GSC1*) and *FKS2* (a.k.a. *GSC2*), and mutations in both have been shown to cause echinocandin resistance (4, 11, 12). The third GS subunit paralog, *FKS3*, has not been implicated in resistance and appears to be minimally expressed (11, 13). There is strong evidence for functional redundancy between *FKS1* and *FKS2*, as deletions of both genes are synthetically lethal in both *C. glabrata* and *S. cerevisiae* (13, 14). Interestingly and for reasons that are not yet clear, in *C. glabrata,* clinical echinocandin resistance is associated with mutations in *FKS2* at least twice as often as with mutations in *FKS1* (8, 12, 15). Furthermore, many questions still remain about the regulation of GS expression in *C. glabrata* and the interplay between *FKS1* and *FKS2* expression in culture and in the host.

Although *FKS1* and *FKS2* are functionally redundant, evidence of their differential regulation has been reported in both *C. glabrata* and *S. cerevisiae*. For instance, in *S. cerevisiae, FKS1* was reported to be highly expressed during vegetative growth, whereas *FKS2* is normally expressed at low levels but upregulated by mating pheromone, calcium signaling, or under conditions of stress (e.g., glucose starvation) (14, 16). In both *C. glabrata* and *S. cerevisiae*, expression of *FKS2* but not *FKS1* was shown to be sensitive to either chemical or genetic inhibition of the calcineurin pathway (13, 14, 16). It has also been shown that deletion of *FKS1* induces a strong compensatory upregulation of *FKS2*, but not vice versa (17). Comparisons of *FKS1* and *FKS2* expression in *C. glabrata* during vegetative growth in culture have yielded a wide range of results. Although some of those studies have reported their findings solely as *FKS1/FKS2* expression ratios but not their individual expression levels, somewhat limiting potential interpretations, the published *FKS1*/*FKS2* expression ratios range from 0.5 (11) to 5.9 (13). These results, along with evidence of the regulation of *S. cerevisiae FKS2* by environmental stress, suggest that both absolute and relative expressions of the two GS subunits can vary widely, depending on culture conditions and environment, but this has not been extensively studied in *C. glabrata*. Finally, using cell cycle synchronization by the mating pheromone α-factor, it has been shown that in *S. cerevisiae, FKS1* transcript abundance is regulated by the cell cycle, peaking in S-phase, consistent with GS being necessary for building the cell wall of the growing bud (14). However, the corresponding experiment could not be done for *FKS2* because its expression in cells released from α-factor arrest into a glucose medium was undetectably low. Because *C. glabrata* does not respond to mating pheromones and cannot be thus synchronized, no information is currently available about cell cycle regulation of GS expression in this fungus.

Most studies of GS subunit expression in *C. glabrata* have relied on measuring the abundance of *FKS1* and *FKS2* mRNAs by quantitative reverse transcriptase PCR (qRT-PCR), which does not distinguish between transcriptional and post-transcriptional regulation. On the other hand, methods to specifically examine promoter activity, for example, via the use of transcriptional reporters, have not been applied to these genes. Furthermore, most studies have measured *FKS1* and *FKS2* mRNA abundance by qRT-PCR using a single primer pair in the very 5′ end of the ORF (11, 13, 17–19), which can also miss the effects of post-transcriptional regulation by not scrutinizing the complete transcripts. Indeed, there is some evidence of post-transcriptional regulation of GS expression in *C. glabrata*, as *FKS1* mRNA and protein levels were shown to be affected by Ssd1, a factor that binds certain mRNAs and regulates their translation (17). Finally, only a very limited analysis of how GS protein expression correlates with corresponding transcript abundance has been reported (17).

In this study, we employed a combination of approaches, including time-lapse imaging, qRT-PCR, flow cytometry, and mining of publicly available RNAseq data sets, to uncover new aspects of the regulation of *FKS1* and *FKS2* expressions in *C. glabrata*. Our results showed that transcription of both *C. glabrata FKS1* and *FKS2* is regulated by the cell cycle, peaking in S-phase, that calcineurin-mediated upregulation of *FKS2* expression in the *fks1∆* mutant is not accompanied by increased *FKS2* promoter activity and therefore occurs at the post-transcriptional level, and that although under normal vegetative growth conditions, *FKS1* is expressed at much higher levels than *FKS2*, under conditions of host-imposed stress, *FKS2* is expressed at equivalent or higher levels to *FKS1*.

## RESULTS

### Promoter activity of both *FKS1* and *FKS2* is regulated by the cell cycle

**Fig 1:**
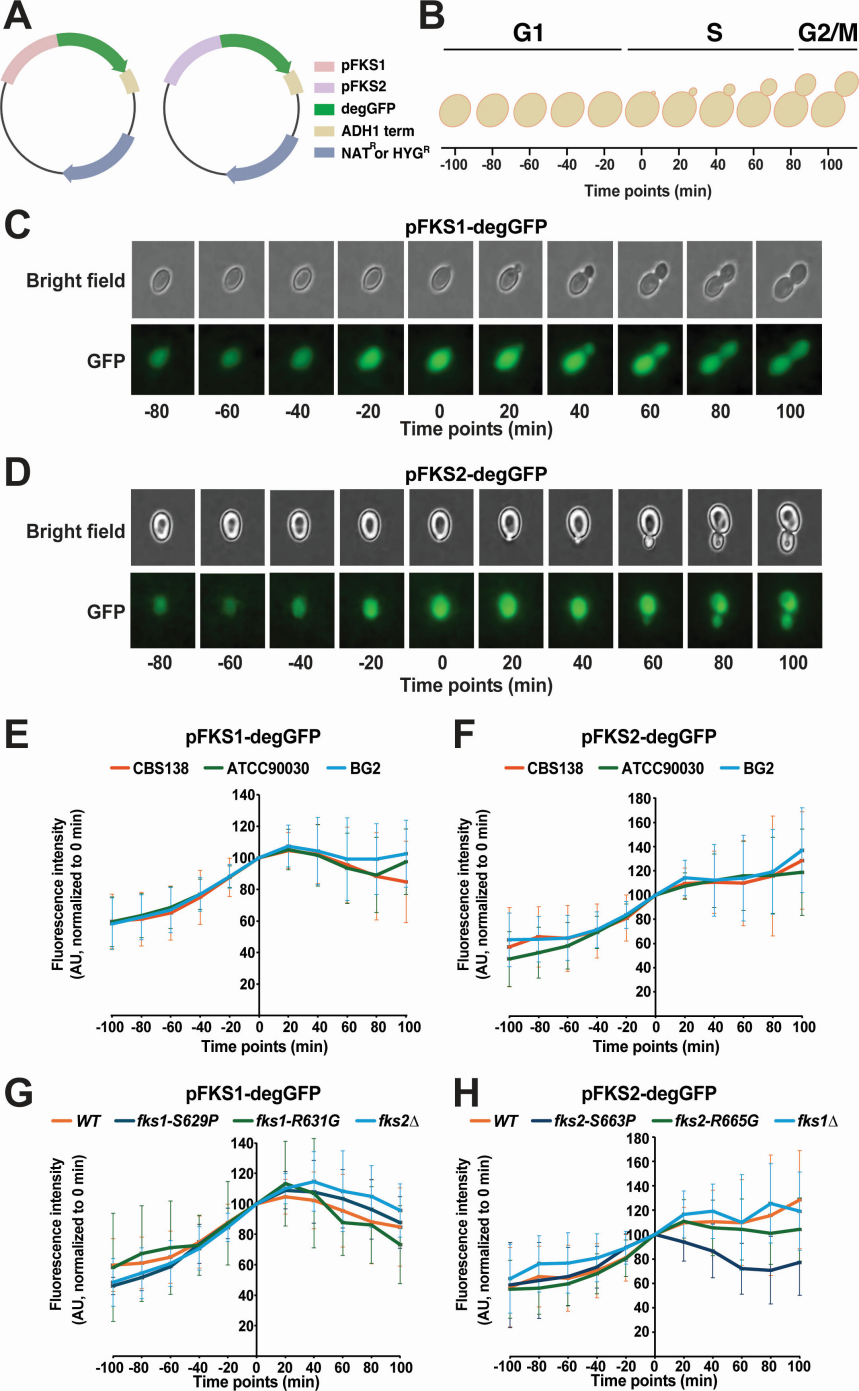
DegGFP transcriptional reporter reveals cell cycle regulation of *FKS1* and *FKS2* promoters. (A) Schematic representation of the plasmids carrying p*FKS1-degGFP* and p*FKS2-degGFP*. (B) Schematic representation of the cell cycle progression in budding yeast. (C) A representative time-lapse image progression of p*FKS1-degGFP*. (D) A representative time-lapse image progression of p*FKS2-degGFP*. (E) p*FKS1* activity peaks in early S-phase. Average *pFKS1-degGFP* fluorescence intensities over 200 min of time-lapse microscopy of strains CBS138 (*n* = 76), ATCC90030 (*n* = 46), and BG2 (*n* = 33) are shown. (F) p*FKS2* activity rises throughout G1 and stays elevated in S-phase. Average p*FKS2-degGFP* fluorescence intensities over 200 min of time-lapse microscopy of strains CBS138 (*n* = 35), ATCC90030 (*n* = 33), and BG2 (*n* = 53) are shown. (G) Echinocandin-resistant *fks1* mutants S629P (*n* = 9) and R631G (*n* = 29) do not affect p*FKS1* cell cycle regulation. Both mutants were made in the CBS138 background, and the WT curve is the same as the CBS138 curve in panel E. (H) Echinocandin-resistant *fks2* mutants S663P (*n* = 31) and R665G (*n* = 33) do not significantly affect p*FKS2* cell cycle regulation, although p*FKS2-degGFP* fluorescent signal is noisier than p*FKS1-degGFP* fluorescent signal. Both mutants were made in the CBS138 background, and the WT curve is the same as the CBS138 curve in panel F. In panels E–H, the fluorescence intensity of every cell at every time point was normalized to that of time point 0 for that cell and shown as a percentage.

Because cell cycle synchronization is not readily achievable in *C. glabrata*, we used a transcriptional reporter approach to determine whether *FKS1* and *FKS2* expression is regulated in a cell cycle-dependent manner. We created plasmids carrying the gene encoding degron-fused GFP (degGFP) driven by either the *FKS1* or *FKS2* promoter (p*FKS1* or p*FKS2*, respectively; Fig. 1A). DegGFP, a short-lived variant of GFP with a half-life of 7 min, was originally created to serve as a transcriptional reporter in yeast to avoid potential artifactual results based on the significantly longer-lived traditional GFP variants (20). Next, asynchronously growing cells carrying the reporter cassettes were embedded in the synthetic complete (SC) medium with 1% low melting agarose, and time-lapse fluorescence microscopy was performed. Multiple fields were imaged for 200 min at 20-min intervals (Fig. 1B), and total fluorescence levels of multiple cells were quantified at every time point relative to the background using NIS-elements software (Nikon Instruments Inc.). Cell cycle stages were defined using the standard budding yeast scheme where unbudded cells are considered to be in G1 phase and bud emergence corresponds to entry into S-phase (21) (Fig. 1B). Examples of representative images are shown in Fig. 1C (p*FKS1-degGFP*) and Fig. 1D (p*FKS2-degGFP*). This approach allowed us to gauge the activity of p*FKS1* and p*FKS2* through the *C. glabrata* cell cycle (from G1, through S, and to G2/M).

We found that both p*FKS1* and p*FKS2* activities increased through the G1 phase (Fig. 1E and F). p*FKS1* activity peaked in the early S phase (20 min time point) and then tended to decrease as cells progressed toward and through G2 (Fig. 1E). In contrast, p*FKS2* activity tended to stay constant or even increase through S and G2 (Fig. 1F). However, the p*FKS2-degGFP* signal was more variable or noisy than the p*FKS1-degGFP* signal, possibly due to the fact that the fluorescent signal from p*FKS2* was significantly weaker than from p*FKS1* (see below). To verify that these patterns were reproducible in other *C. glabrata* strain backgrounds, we transformed the reporter plasmids into two other commonly used strains, ATCC90030 and BG2. Although differences among individual cells produced experimental variation in all strain backgrounds, similar p*FKS1* and p*FKS2* activity patterns throughout the cell cycle were observed in all strains (Fig. 1E and F).

Next, we examined whether mutations in β-glucan synthase affected *FKS1* and *FKS2* promoter activity throughout the cell cycle. We used CRISPR-Cas9 to construct chromosomal mutations in the hot-spot regions of Fks1 (S629P and R631G) and Fks2 (S663P and R665G), which are associated with clinical echinocandin resistance (6, 11, 12, 15, 22). We also asked whether the activity of *FKS1* and *FKS2* promoters through the cell cycle would be affected by the loss of the other β-glucan synthase subunit (i.e., whether the activity of p*FKS1* would be affected by *fks2∆* and vice versa). To this end, chromosomal deletions of *FKS1* and *FKS2* open reading frames (ORF) were created separately, and the p*FKS1* reporter was transformed into the *fks2∆* strain, whereas the p*FKS2* reporter was transformed into the *fks1∆* strain. All mutants containing reporter plasmids were analyzed by time-lapse microscopy, as described above. Although all strains produced some cell-to-cell variability, we found that none of the mutants strongly changed the temporal dynamics of *FKS1* or *FKS2* promoter activity throughout the cell cycle (Fig. 1G and H). Together, these experiments showed that both *FKS1* and *FKS2* promoters are regulated by the cell cycle, with the highest expression observed during the S phase, consistent with the role of β-glucan synthase in building the cell wall of the growing bud.

### *C. glabrata FKS1* is expressed at higher levels than *FKS2*

**Fig 2:**
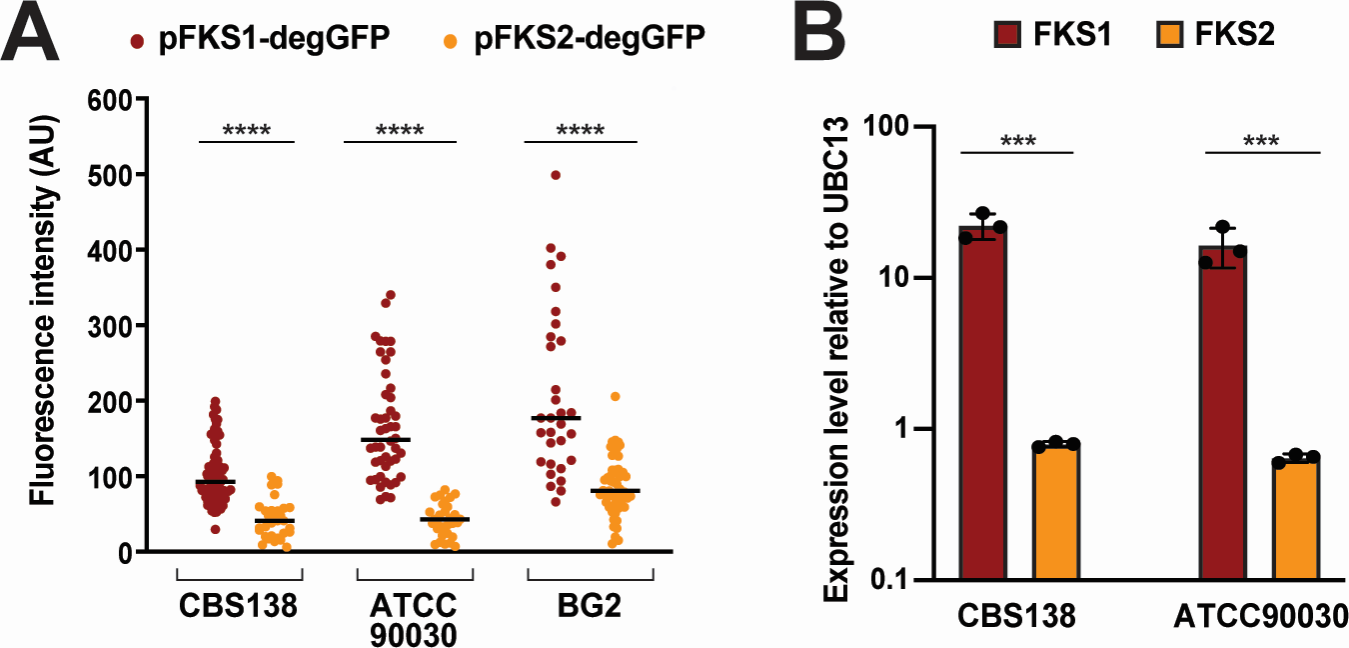
Under standard culture conditions, *FKS1* promoter activity and mRNA levels are higher than those of *FKS2*. (A) p*FKS1-degGFP* produces a stronger fluorescent signal than p*FKS2-degGFP* in three different genetic backgrounds. Shown are fluorescence intensities at time 0 of every cell analyzed during time-lapse microscopy (Fig. 1). One dot represents one cell. (B) qRT-PCR showed that *C. glabrata* cells growing exponentially in YPD contain significantly more *FKS1* mRNA than *FKS2* mRNA. Statistical significance was determined using an unpaired *t*-test for normally distributed data and the Mann-Whitney test for non-normally distributed data. ***P < 0.001; ****P < 0.0001.

During the microscopy experiments, we observed that p*FKS1-degGFP* produced significantly higher fluorescence levels than p*FKS2-degGFP* in all strains (Fig. 2A), suggesting that the *FKS1* gene has stronger promoter activity under our experimental conditions (i.e., in cells cultured on agarose pads containing SC growth medium). However, Garcia-Effron et al. previously reported the results of quantitative reverse transcriptase PCR (qRT-PCR) experiments showing that in culture, *C. glabrata FKS2* expression was greater than *FKS1* (11). This was in contrast to results from *Saccharomyces cerevisiae*, where *FKS1* is expressed at higher levels than *FKS2* in culture (14). Because of this inconsistency, we measured *FKS1* and *FKS2* expression by qRT-PCR in ATCC2001 and ATCC90030 strains cultured in liquid YPD. In both strains, *FKS1* expression was significantly higher than that of *FKS2*, consistent with the microscopy results (Fig. 2B). To understand the source of the disagreement, we closely examined the primer sequences in the previous study (see Table 2 in Reference [23]) and discovered that in that study, primers assigned to *FKS1* were in fact complementary to *FKS2* and vice versa. Once the mistake was uncovered, the qRT-PCR results from Garcia-Effron et al. (11) and our current study aligned, showing that under laboratory culture conditions, in *C. glabrata*, like in *S. cerevisiae*, *FKS1* is expressed at higher levels than *FKS2*, which correlated well with the stronger *FKS1* promoter activity observed by microscopy.

### *FKS2* expression is somewhat sensitive to mutations in β-glucan-synthase

Previously reported qRT-PCR experiments have shown that deletion of *FKS1* results in a compensatory increase in *FKS2* expression (17), indicating that expression of GS genes may be sensitive to perturbations in β-glucan-synthase activity. Our fluorescent reporter system has enabled us to ask whether the *FKS2* promoter is more active in the *fks1∆* mutant and vice versa. We also asked whether echinocandin-resistant mutations in Fks1 (*fks1-S629P* and *fks1-R631G*) affected p*FKS1* activity and, similarly, whether echinocandin-resistant mutations in Fks2 (*fks2-S663P* and *fks2-R665G*) affected p*FKS2* activity (e.g., via a feedback loop). Absolute GFP signal intensity (relative to background) was measured in each cell at time 0 (Fig. 1B), that is, the beginning of the S-phase (i.e., the point in the yeast cell cycle known as START) because that was the time point that was the easiest to identify and standardize among different cells. In a complementary approach, we used flow cytometry to measure GFP levels in the same strains, cultured in YPD, using a standard doublet-excluding gating scheme and propidium iodide (PI) staining to exclude dead cells (Fig. S1). Finally, in a parallel series of experiments, we collected total RNA from each of the mutant strains (not carrying the degGFP reporter plasmids) along with the wild-type control, cultured in YPD, and measured *FKS1* and *FKS2* expressions by qRT-PCR.

**Fig 3:**
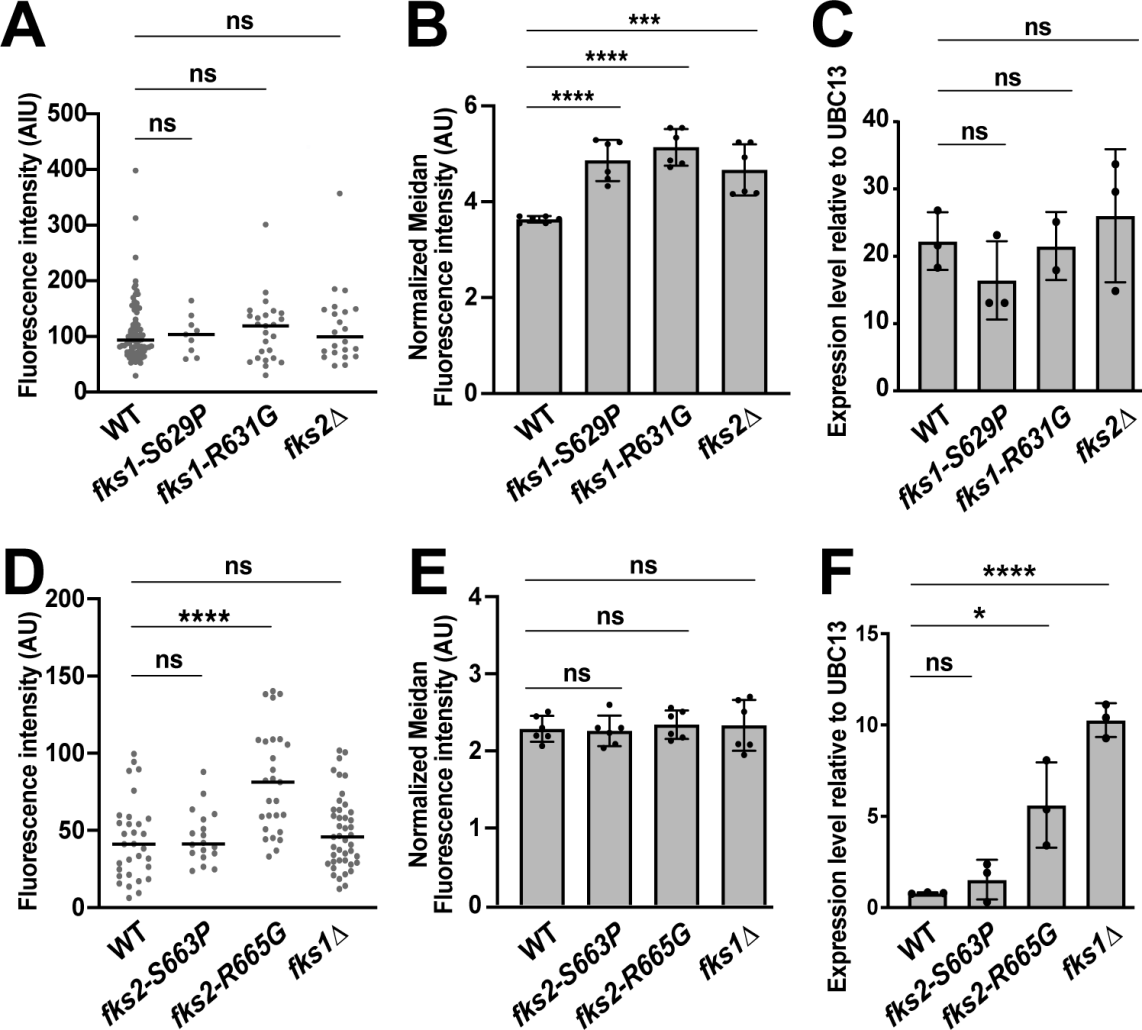
Effects of *fks1* and *fks2* mutations on their promoter activity and mRNA levels. (A) Analysis of fluorescence intensities of single cells at time 0 revealed no difference between p*FKS1-degGFP* activity in wild-type cells, echinocandin-resistant *fks1* mutant cells, or *fks2∆* cells. (B) Flow cytometry analysis indicated an increase in p*FKS1-degGFP* activity in two *fks1* echinocandin-resistant mutants and the *fks2∆* strain relative to the wild-type strain. Flow cytometry data from every strain were normalized relative to a non-fluorescent control and presented as arbitrary units (AU). (C) qRT-PCR analysis of *FKS1* mRNA showed no difference between the wild-type strain and the three mutants. (D) Analysis of fluorescence intensities of single cells at time 0 revealed no difference between p*FKS2-degGFP* activity in wild-type, *fks2-S663P*, and *fks1∆* cells, but an increase in *fks2-R665G* cells. (E) Flow cytometry analysis indicated no difference between p*FKS2-degGFP* activity in wild-type, *fks2-S663P*, and *fks2-R665G* cells, but a slight increase in *fks1∆* cells. Flow cytometry data from every strain were normalized relative to a non-fluorescent control and presented as arbitrary units (AU). (F) qRT-PCR analysis of *FKS2* mRNA showed no difference between the wild-type and the *fks2-S663P* strain, a moderate increase in the *fks2-R665G* strain, and a strong increase in the *fks1∆* strain. Cells were cultured in SC medium for microscopy (A and D) and YPD medium for flow cytometry (B and E) and mRNA isolation (C and F). Statistical significance was determined using an unpaired *t*-test for normally distributed data and the Mann-Whitney test for non-normally distributed data. *P < 0.05; ****P < 0.0001.

The three approaches produced some concordant results and also some differences. First, microscopy analysis of p*FKS1-degGFP* cells and qRT-PCR of *FKS1* both showed no significant differences in *FKS1* promoter strength or mRNA levels, respectively, in *fks1-S629P*, *fks1-R631G*, or *fks2∆* strains (Fig. 3A and C). On the other hand, flow cytometry of p*FKS1-degGFP* cells showed that all three mutant strains had higher levels of degGFP expression than the control strain (Fig. 3B). It is not clear whether this difference is attributable to the differences in the cell cycle distributions of the analyzed populations, different sensitivities of microscopy vs. flow cytometry, different medium composition, or a combination of these factors. Analysis of the *FKS2* promoter strength and expression by the different methods also produced some intriguing similarities and discrepancies. First, all three methods showed no difference between the expression of *fks2-S629P* and WT *FKS2* (Fig. 3D through F). In contrast, the *fks2-S665G* mutation caused an increase in p*FKS2* promoter activity, as seen by fluorescent microscopy (Fig. 3D), and in *FKS2* mRNA levels, as determined by qRT-PCR (Fig. 3F), suggesting that this *fks2* mutation caused a compensatory increase in *FKS2* expression via its promoter upregulation. Interestingly, examination of the *fks1∆* mutant revealed a striking difference between p*FKS2* activity and *FKS2* mRNA levels. Both fluorescent microscopy and flow cytometry showed that p*FKS2* activity is not altered in the *fks1∆* mutant (Fig. 3D and E). In contrast, and consistent with previously published results (17), *FKS2* mRNA levels were increased >10-fold in the *fks1∆* strain (Fig. 3F). This indicated that the effect of *fks1∆* on *FKS2* expression may not be at the level of the promoter and that *FKS2* was likely subject to post-transcriptional regulation in the *fks1∆* mutant.

### Effect of *fks1∆* on *FKS2* expression depends on the growth condition and shows evidence of post-transcriptional regulation

**Fig 4:**
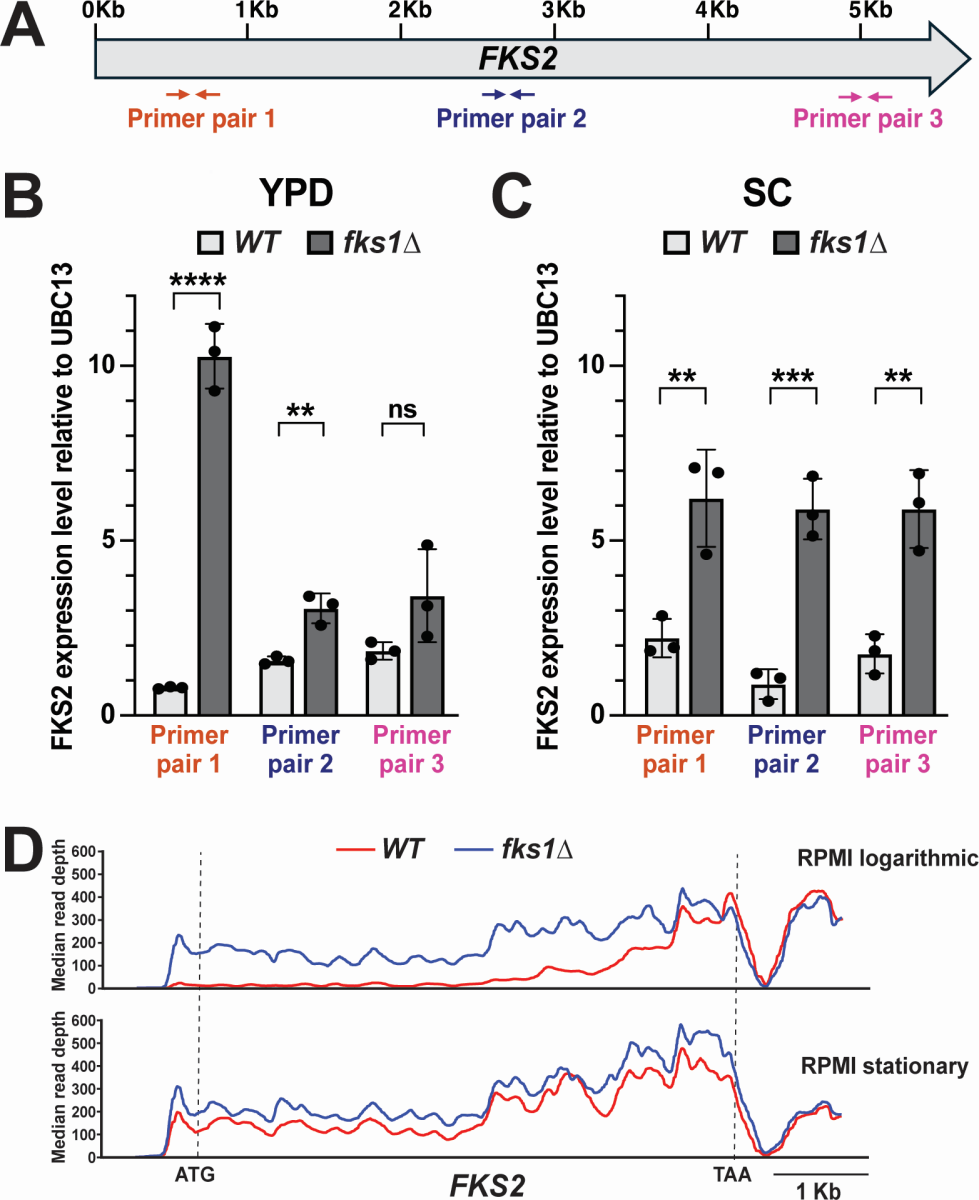
Effect of *fks1∆* on *FKS2* mRNA abundance varies across the *FKS2* ORF and depends on culture conditions. (A) Schematic representation showing the location of the three *FKS2* qRT-PCR primer pairs. (B) In cells cultured in rich medium (YPD), the strong *fks1∆-*driven increase in *FKS2* mRNA is seen only when primer pair 1, which binds at the ORF’s 5′ end, is used. (C) In cells cultured in synthetic complete medium (SC), the *fks1∆* effect on *FKS2* mRNA is more moderate and independent of which primer pair is used. (D) Sashimi plots show that the *fks1∆* effect on *FKS2* transcript levels is stronger in the 5′ end of the gene than the 3′ end of the gene and stronger in log phase cells than in stationary phase cells. The sashimi plots show RNA-seq read depth across the *FKS2* ORF in the wild-type strain and *fks1∆* mutant cultured in RPMI and harvested either in the log or stationary phase. Statistical significance was determined using an unpaired *t*-test. **P < 0.01; ***P < 0.001; ****P < 0.0001.

Both *FKS1* and *FKS2* are large genes, over 5 Kb in length. The primers used by us, as well as primers used in previous studies that examined *FKS2* expression (11, 13, 17–19), hybridized to *FKS2* near the 5′ end of its open reading frame (ORF, Fig. 4A). To look at *FKS2* mRNA levels more broadly, we used two additional primer pairs, hybridizing in the middle and at the 3′ end of the *FKS2* ORF (Fig. 4A). Interestingly, the observed difference between *FKS2* mRNA levels in the WT and *fks1∆* strain drastically depended on primer location. Whereas the 5′ end primer pair indicated that *FKS2* is expressed >10-fold higher in the *fks1∆* mutant than in the WT strain, using the middle primer pair reduced this difference to about 2-fold, whereas using the 3′ primer pair did not produce a statistically significant change (Fig. 4B).

We considered that the difference between *FKS2* promoter activity (observed by fluorescent microscopy) and *FKS2* mRNA levels (observed by qRT-PCR) in the *fks1∆* mutant may be due, in part, to the growth conditions of the two experiments. The biggest difference was that during time-lapse microscopy, the cells were growing on the synthetic complete (SC) medium because it produces less auto-fluorescence than YPD, the standard rich yeast growth medium. In contrast, cells were cultured in YPD for RNA preparation. Thus, it was possible that the *FKS2* promoter was not activated by *fks1∆* in SC but was activated by *fks1∆* in YPD. To test this hypothesis, we isolated RNA from WT and *fks1∆* strains cultured in SC, followed by *FKS2* qRT-PCR. We observed that despite the unchanged p*FKS2* activity in the *fks1∆* mutant cultured in SC (Fig. 3D), *FKS2* mRNA levels were significantly (3-fold to 5-fold) increased in the *fks1∆* mutant also cultured in SC (Fig. 4C). Furthermore, unlike in YPD, this increase was more uniform across the *FKS2* ORF, revealing the effect of culture medium on *FKS2* mRNA processing in the *fks1∆* mutant.

As part of a separate, as-yet-unpublished study, we generated RNA-seq data for CBS138 and an isogenic *fks1∆* strain. The strains were cultured in Roswell Park Memorial Institute medium (RPMI1640), which is frequently used to culture mammalian cells and perform standardized fungal drug sensitivity testing, and one biological replicate was analyzed for each strain. This gave us an opportunity to examine the effect of *fks1∆* on *FKS2* RNA abundance across its entire transcript. To this end, we generated sashimi plots of *FKS2* in the WT and *fks1∆* mutant strains harvested either during stationary (overnight culture) or logarithmic growth stages in RPMI. These sashimi plots produced several insights consistent with the qRT-PCR results obtained above. First, we observed that the difference between *FKS2* transcript abundance in WT and *fks1∆* cells was not uniform across the gene’s ORF. In logarithmically growing cells, this abundance was much higher in *fks1∆* than in WT cells at the 5′ end of the gene, but this difference gradually decreased toward the 3′ end of the gene (Fig. 4D), consistent with qRT-PCR results obtained in YPD-cultured cells (Fig. 4B). In contrast, in stationary cells, the difference in *FKS2* transcript abundance between WT and *fks1∆* cells was both much smaller and more uniform throughout the ORF (Fig. 4D), similar to qRT-PCR results obtained with SC-cultured cells (Fig. 4C). Together, the results of all these experiments showed that the effect of *fks1∆* on *FKS2* expression depends on the growth conditions, is not accompanied by increased *FKS2* promoter activity, and is not uniform across the *FKS2* ORF, providing evidence of post-transcriptional regulation.

### FK506 affects *FKS2* expression differently in WT and *fks1∆* cells

**Fig 5:**
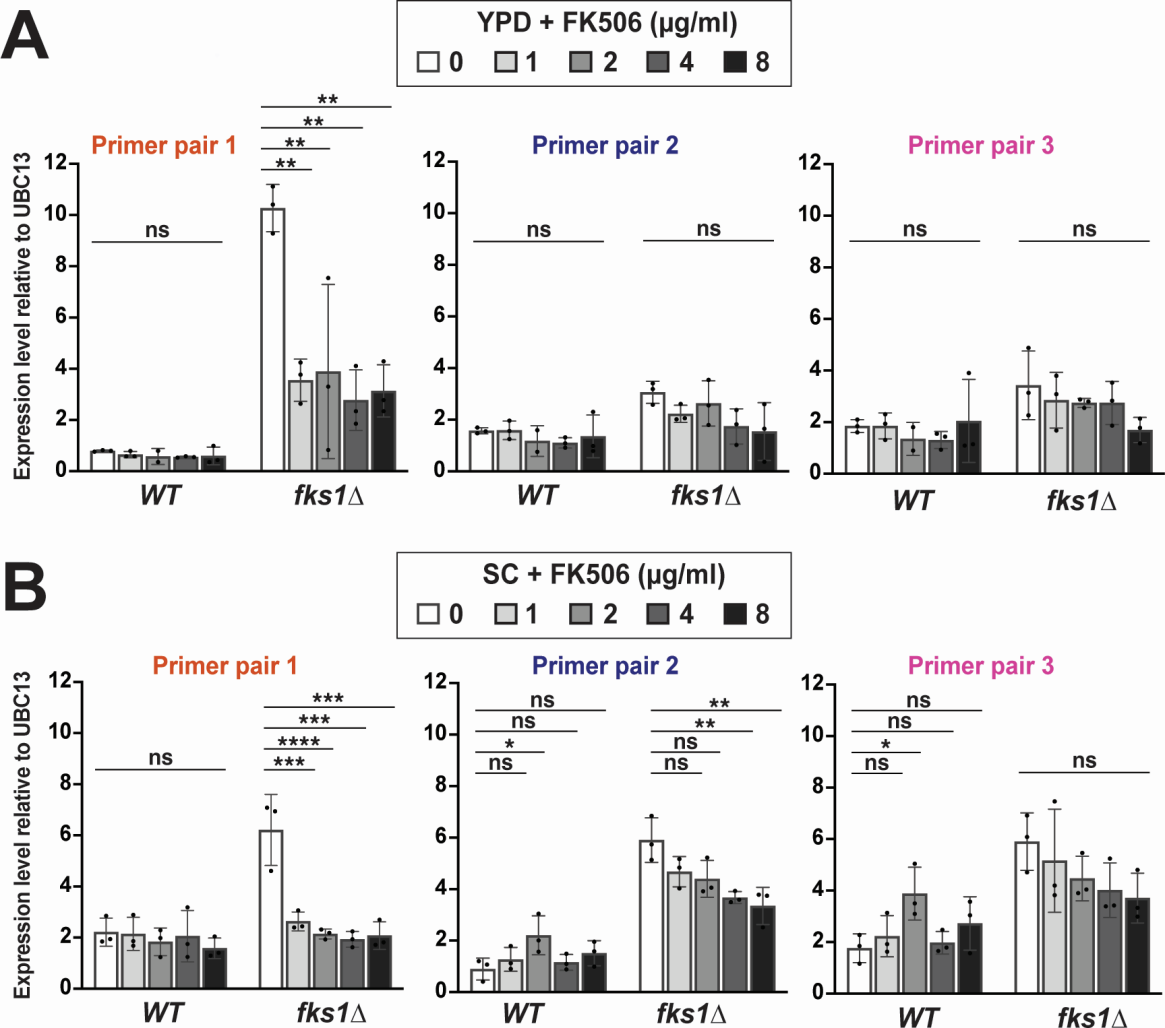
Inhibition of calcineurin signaling by FK506 suppresses *FKS2* transcription in the *fks1∆* mutant in a dose-dependent manner. (A) qRT-PCR of *FKS2* using the three different primer pairs (Fig. 4A) in YPD-cultured cells in the presence of FK506. (B) qRT-PCR of *FKS2* using the three different primer pairs (Fig. 4A) in SC-cultured cells in the presence of FK506. Statistical significance was determined using an unpaired *t*-test. *P < 0.05; **P < 0.01; ***P < 0.001; ****P < 0.0001.

FK506 is a small molecule inhibitor of the calcineurin pathway, which regulates *FKS2* expression (13, 14). It has been reported that *fks1∆* cells are highly sensitive to FK506, presumably because FK506 inhibits *FKS2* expression, leaving the cell without a sufficient amount of functional β-glucan synthase (13, 17). Nevertheless, direct evidence for FK506-mediated inhibition of *FKS2* expression in the WT cells is scarce. We used qRT-PCR with all three primer pairs (Fig. 4A) to measure *FKS2* expression in WT and *fks1∆* strains cultured either in YPD or in SC. Similar results were obtained with the two different culture media. First, we observed that in WT cells carrying functional *FKS1*, the addition of FK506 (1, 2, 4, or 8 µg/mL) had little to no effect on *FKS2* expression either in YPD or in SC, regardless of which primer pair was used (Fig. 5A and B). In contrast, and consistent with the current paradigm, the addition of FK506 strongly reduced *FKS2* transcription in *fks1∆* cells. This was particularly evident using the 5′ end primers on RNA derived from YPD-cultured samples (Fig. 5A) and when using all three primer pairs on RNA derived from SC-cultured samples (Fig. 5B). Furthermore, FK506 showed evidence of dose-dependent suppression of *FKS2* transcription in *fks1∆* cells, particularly in SC-derived samples (Fig. 5B). Finally, we also measured *FKS1* expression in the presence of FK506, both in WT and *fks2∆* strains, and observed no significant differences (Fig. S2). Together, these experiments indicated that inhibition of the calcineurin pathway by FK506 significantly reduces *FKS2* expression in the absence of functional *FKS1* but not in WT cells.

### Fks2 protein levels are affected by *fks1∆* and FK506

Given the discrepancies between the effects of *fks1∆* and FK506 on transcripts originating from different parts of the *FKS2* ORF, particularly in YPD-cultured cells, we sought to examine the effects of *fks1∆* and FK506 on the ultimate product of *FKS2* gene expression, the Fks2 protein. To this end, we fused the gene encoding the HaloTag (HT) (24) to the N-terminus of the endogenous chromosomal *FKS2*, creating HT-Fks2. Because *fks2∆* does not have a strong mutant phenotype, instead of a standard complementation assay, we used a different genetic strategy to test whether this construct was fully functional. This strategy relied on the fact that the *fks2-S663P* mutant confers strong echinocandin resistance and that the gene must be expressed for this resistance to occur, as *fks2∆* cells are not echinocandin-resistant (13). Thus, we also tagged the *fks2-S663P* mutant with the HaloTag and asked whether echinocandin resistance was preserved. We found that adding the HaloTag to the N-terminus of Fks2^S663P^ protein did not reduce its ability to cause caspofungin resistance (Fig. S3), indicating that HT-Fks2 is expressed and functional.

**Fig 6:**
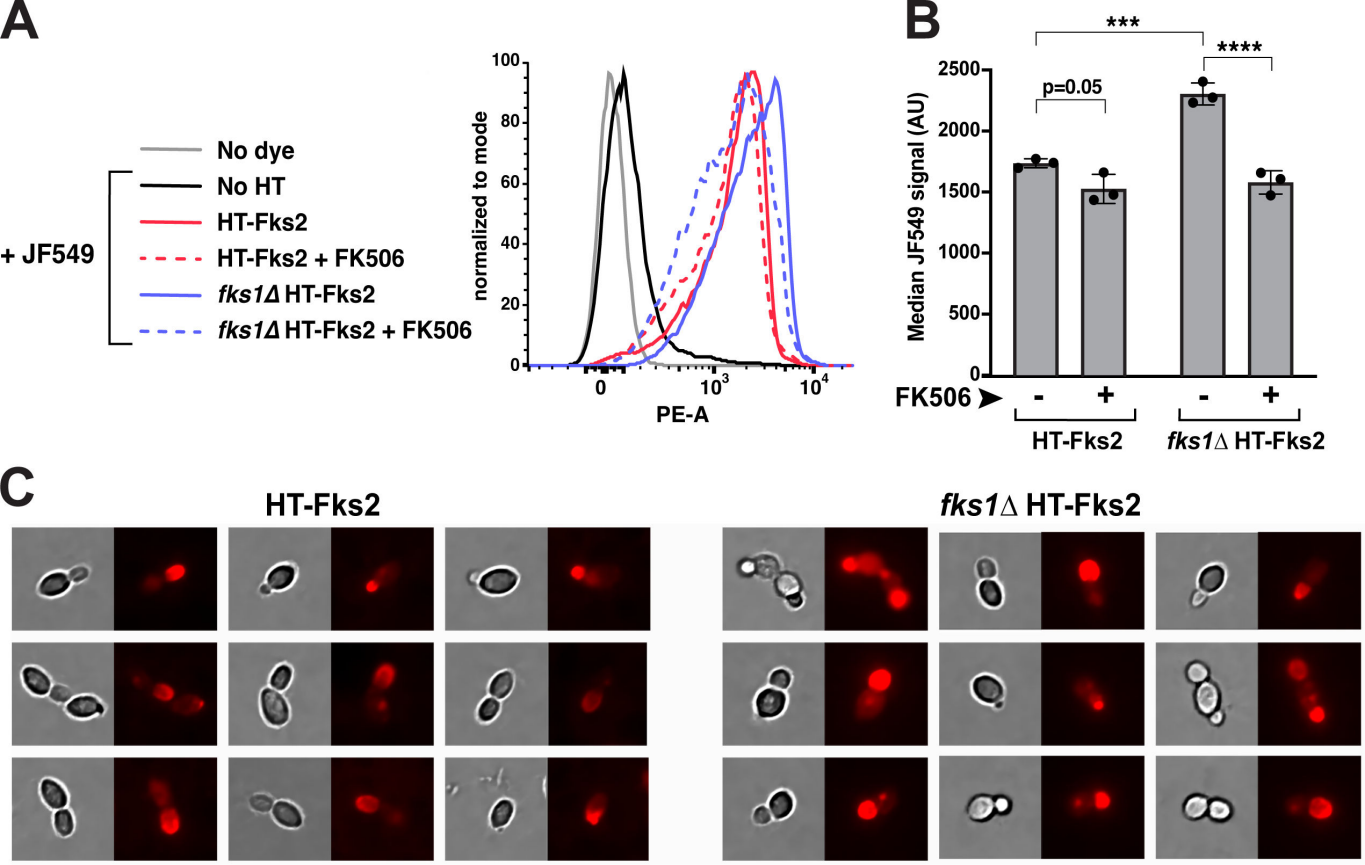
Effects of *fks1∆* and calcineurin inhibitor FK506 on Fks2 protein. (A) Flow cytometry was used to measure the levels of HaloTag-Fks2 (HT-Fks2) bound to Janelia Fluor 549 (JF549). A representative flow cytometry result from the different strains and controls is shown. (B) Quantification of flow cytometry results showed that HT-Fks2 protein increase in the *fks1∆* strain was fully abolished by FK506 (2 µg/mL). (C) Fluorescence microscopy confirmed the increase in HT-Fks2 protein level in the *fks1∆* strain and also showed that in the wild-type strain, HT-Fks2 was localized to the growing bud tip, whereas overexpressed HT-Fks2 in the *fks1∆* mutant was present more diffusely throughout the daughter cell. Statistical significance was determined using an unpaired *t*-test. ***P < 0.001; ****P < 0.0001.

To examine the effects of *fks1∆* and FK506 on HT-Fks2 expression, we incubated YPD-grown cells (WT or *fks1∆*, with or without 2 µg/mL FK506) with fluorescent Janelia Fluor HaloTag Ligand 549 (JF549), followed by flow cytometry analysis (Fig. 6A). These experiments showed that HT-Fks2 protein levels were significantly increased in the *fks1∆* mutant, but that this increase was not high (approximately 30%, Fig. 6B), which is more in line with the effects of *fks1∆* on *FKS2* transcript towards the middle and 3′ end of the gene rather than its 5′ end (Fig. 4B). Furthermore, we observed that FK506 treatment not only significantly reduced HT-Fks2 abundance in *fks1∆* cells, which is consistent with its effects on *FKS2* transcripts, but that it also slightly reduced HT-Fks2 abundance in WT cells (Fig. 6B). Thus, inhibition of the calcineurin pathway in *C. glabrata* fully abrogates the elevated expression of Fks2 protein in the absence of Fks1.

Finally, to investigate whether the loss of *FKS1* affects not just the abundance but the localization of Fks2, we used JF549 to visualize HT-Fks2 by fluorescent microscopy. We observed that in WT cells, HT-Fks2 was predominantly localized to the growing bud (daughter cell) and that in larger daughter cells, HT-Fks2 was concentrated near the bud tip, consistent with Fks2's role in building the growing cell wall (Fig. 6C). In *fks1∆* cells, consistent with the flow cytometry results (Fig. 6B), we observed a stronger overall fluorescent signal, as well as increased HT-Fks2 localization throughout the daughter cell (Fig. 6C), confirming that in *fks1∆* cells, Fks2 protein was produced at higher levels and showing that it was not always appropriately targeted to the sites of new cell wall production.

### Evidence for increased *FKS2* expression in host tissues

Thus far, we have examined *FKS1* and *FKS2* expression in culture and found that in actively growing cells, the *FKS1* promoter was more active than the *FKS2* promoter, and *FKS1* was expressed at significantly higher levels than *FKS2* (Fig. 2). This was consistent with their relative expression in *S. cerevisiae* during unstressed vegetative growth; however, in *S. cerevisiae, FKS2* expression is induced by glucose starvation (14). We also observed that environmental conditions affected *FKS2* expression (e.g., compare 4C [SC medium] with 4B [YPD medium]) and that *FKS2* is expressed more abundantly in stationary phase cells than in logarithmically growing cells (Fig. 4D). Unlike for *S. cerevisiae*, the physiologically relevant growth environment for *C. glabrata* is host tissues, where the fungus is likely exposed to a number of stresses, including glucose limitation (25). We recently reported RNA sequencing (RNA-seq) of *C. glabrata* infecting a macrophage-like cell line (THP-1) (26), whereas another recent study performed dual RNA-seq of *C. glabrata-*infected mouse kidneys (27). To gain an understanding of *FKS1* and *FKS2* expression *in vivo*, we extracted total *FKS1* and *FKS2* read counts from both our study (26) and the mouse kidney infection study (27).

**Fig 7:**
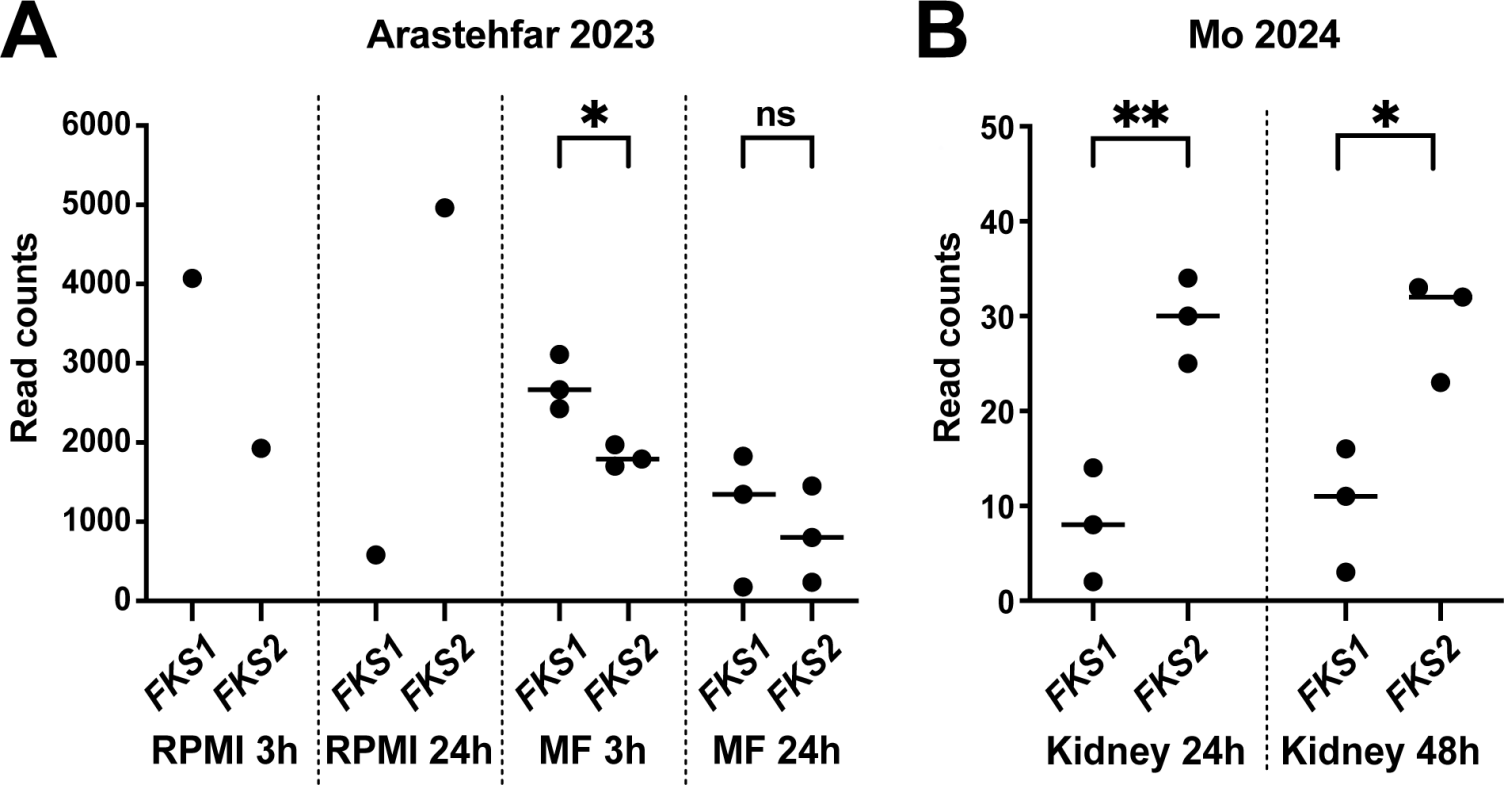
In *C. glabrata* cells growing under host conditions, *FKS2* is expressed at similar or higher levels than *FKS1*. (A) *FKS1* and *FKS2* read counts were extracted from the RNA-seq data set of *C. glabrata* cultured in RPMI or infecting the macrophage-like THP-1 cell line (26). (B) *FKS1* and *FKS2* read counts were extracted from the RNA-seq data set of *C. glabrata* infecting mouse kidneys (27). Statistical significance was determined using an unpaired *t*-test. *P < 0.05; **P < 0.01.

In our study, *C. glabrata* transcriptomes were analyzed under conditions of *in vitro* culture (RPMI), both in log phase (3 h) and stationary phase (24 h) cells, as well as 3 h and 24 h after infection of THP-1 cells. Mining of *FKS1* and *FKS2* expression data produced conclusions that were remarkably consistent with expectations. In the log phase culture, *FKS1* expression was over 2-fold higher than *FKS2*, whereas in the stationary phase, cell *FKS2* expression was over 8-fold higher than *FKS1* (Fig. 7A). Because only one sample was analyzed for each *in vitro* growth condition, no information on the variation within the data was available; hence, the true differences in expression may somewhat differ from these numbers. In the macrophage environment, where three biological replicates were analyzed per timepoint, *FKS1* produced about 50% higher read counts than *FKS2* at 3 h post-infection, and no significant difference between the two genes was detected at 24 h post-infection (Fig. 7A), indicating that *FKS1* and *FKS2* were expressed at similar levels in THP-1-infecting *C. glabrata*. Mining of the infected kidney dual RNA-seq data set (27) showed that, first, as expected, the total *C. glabrata* read numbers were significantly lower, likely because fungal RNA constituted a small fraction of the total material analyzed by RNA-seq. However, both *FKS1* and *FKS2* reads were identified, and interestingly, in *C. glabrata*-infected kidneys, *FKS2* reads outnumbered *FKS1* reads by about 3-fold both at 24 h and 48 h post-infection (Fig. 7B). Together, these analyses revealed that within the context of host environmental stresses, *FKS1* and *FKS2* are either expressed equivalently or *FKS2* may be the predominantly expressed GS subunit.

## DISCUSSION

**Fig 8:**
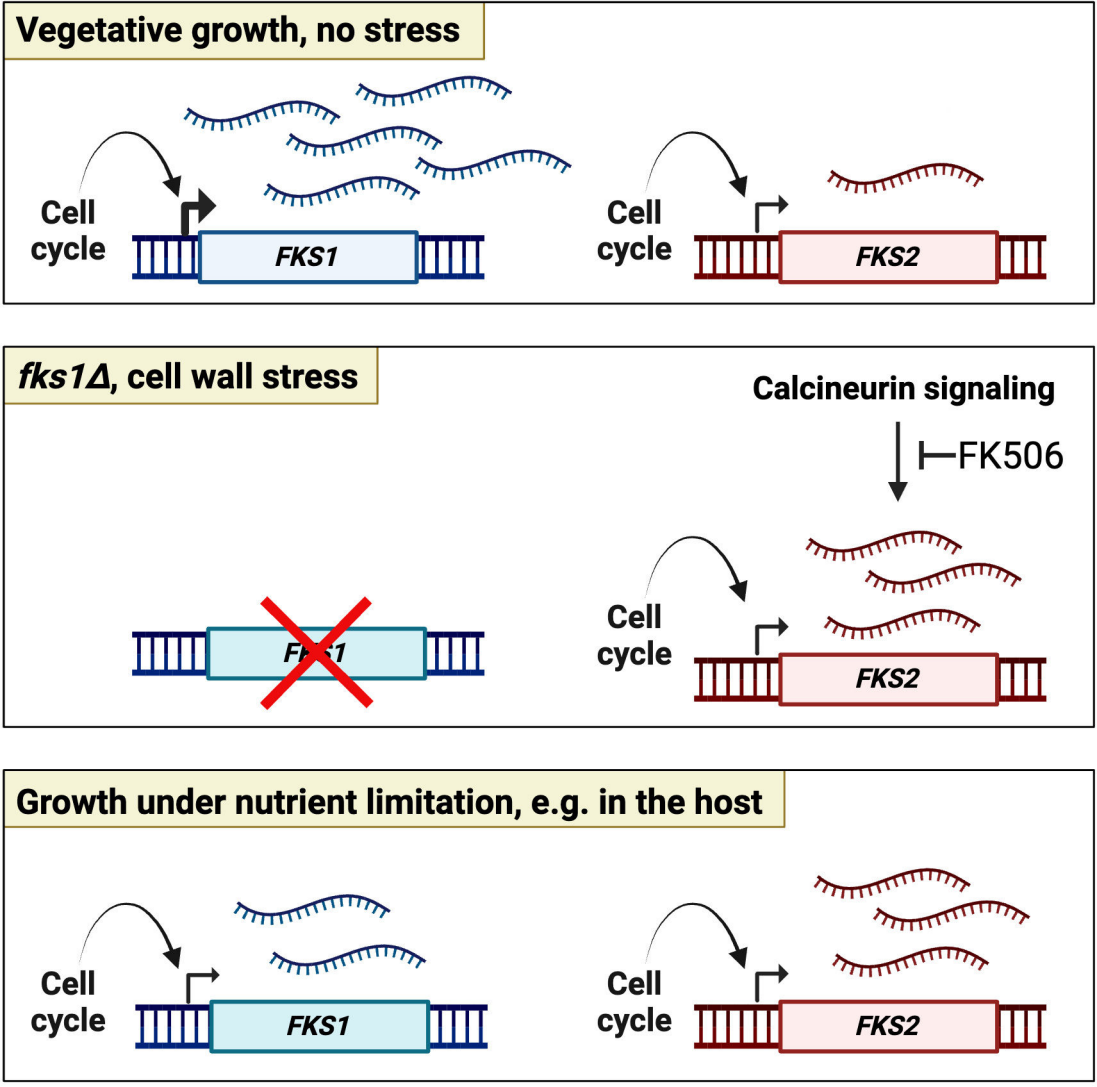
Schematic representation of the key findings of this study. Under unstressed conditions of vegetative growth, both *FKS1* and *FKS2* are regulated transcriptionally by the cell cycle, and *FKS1* is expressed at higher levels than *FKS2*, at least in part due to stronger promoter activation. Under conditions of cell wall stress, such as the *fks1∆* mutant, *FKS2* mRNA levels are increased as a result of calcineurin signaling, but this effect is not accompanied by a commensurate increase in *FKS2* promoter activity, indicating post-transcriptional regulation. Finally, under conditions of glucose limitation, such as in the host, *FKS2* is expressed at similar or higher levels than *FKS1*. The figure was made using BioRender (institutional license).

In this study, we used a combination of approaches to examine the regulation of expression of *FKS1* and *FKS2* genes in *C. glabrata*. Employing a short-lived variant of GFP, degGFP, as a transcriptional reporter showed that both *FKS1* and *FKS2* promoter activities are regulated by the cell cycle, peaking in the S-phase. We also found that during normal growth in culture, the *FKS1* promoter is significantly more active than the *FKS2* promoter and that this difference correlates well with a difference in their mRNA abundance determined by qRT-PCR. We also found that neither promoter activity nor mRNA abundance was greatly altered in several *fks1* or *fks2* point mutants causing echinocandin resistance, consistent with a previous report (11). On the other hand, we found that although, consistent with previous reports (17), *FKS2* mRNA levels significantly increased in the *fks1∆* mutant, there was no concomitant increase in *FKS2* promoter activity, suggesting post-transcriptional regulation. We also used qRT-PCR, Fks2 protein analysis, and calcineurin inhibitor FK506 to show that, consistent with previous studies, *fks1∆*-mediated induction of *FKS2* expression requires the calcineurin pathway, culture conditions (e.g., rich vs. defined medium) affect *FKS2* expression, and qRT-PCR using primers binding in the middle and 3′ of the *FKS2* ORF correlated better with Fks2 protein level than using primers binding in the 5′ end of the ORF. Finally, we mined RNA-seq data sets of *C. glabrata* inhabiting macrophages or mouse kidneys to show that, unlike in culture, under conditions of host-imposed environmental stresses, *FKS2* is expressed at similar or higher levels than *FKS1*. Together, these results have revealed that the cell cycle and environmental conditions regulate the expression of the two GS subunits in *C. glabrata*, that this regulation occurs via both transcriptional and post-transcriptional mechanisms, and that *FKS2* may be the predominantly expressed GS subunit *in vivo* (Fig. 8).

Previous studies that have examined *C. glabrata* GS subunit expression (11, 13, 17–19) have all measured *FKS1* and *FKS2* expression using only qRT-PCR with a single pair of primers binding to the very 5′ end of the gene. Although this approach can provide some information about mRNA abundance, it does not elucidate whether any effects on transcript abundance are due to transcriptional or post-transcriptional regulation. In contrast, using qRT-PCR analysis with multiple primer pairs combined with promoter-driven reporters can reveal which modes of transcription regulation are at play. Indeed, our experiments using promoter-degGFP fusions have revealed that cell cycle progression regulates *FKS1* and *FKS2* expression at the level of transcription initiation, with *FKS1* and *FKS2* promoters showing the highest activity in S-phase, consistent with GS requirement for building the cell wall of the growing daughter cell. On the other hand, the induction of *FKS2* in the *fks1∆* mutant via the calcineurin pathway is largely post-transcriptional, as there was no promoter activity difference when assessed either by microscopy or flow cytometry of the p*FKS2-degGFP* fusion, whereas *FKS2* mRNA abundance was about three-fold higher in the *fks1∆* mutant than in the wild-type strain grown in synthetic medium (the same medium as used for microscopy). Therefore, we can conclude that the calcineurin signaling pathway upregulates *FKS2* expression in the *fks1∆* mutant largely post-transcriptionally, for example, via mRNA stabilization. Indeed, it has been shown that in *Cryptococcus neoformans,* calcineurin target Puf4 (homolog of Puf4 and Mpt5 in *S. cerevisiae*) is an RNA-binding protein that stabilizes the mRNA and regulates the expression of *FKS1*, the sole GS subunit gene in *C. neoformans* (28). The accumulation of *FKS2* 5′ mRNA relative to 3′ mRNA in *fks1∆* mutants is also consistent with other modes of post-transcriptional processing, for example, by an RNA nuclease, or with premature transcript termination. Future studies, for example, examining *FKS2* mRNA stability under different conditions, are necessary to distinguish between these possibilities and identify the mechanisms involved.

Our results may help explain several observations relating to clinical echinocandin sensitivity and resistance in *C. glabrata*. Antifungal drug activity is necessarily influenced by the expression of its target. We previously showed that echinocandins (caspofungin and micafungin) are more active against actively dividing cells than cells in the stationary phase (29). Furthermore, echinocandins are significantly less active against *C. glabrata* infecting host organs and tissues, where it likely exists in a slow or non-growing state, than against *C. glabrata* growing in culture (29, 30). Our cell cycle analysis suggests that *C. glabrata* cells in the S-phase, which express the highest amounts of GS but are relatively rare under host conditions, are likely most sensitive to echinocandin inhibition. One limitation of our cell cycle analysis is that the cells carried no nuclear marker; hence, we could not distinguish between the G2 and M phases by following nuclear division, and we also did not follow cells through division back into the G1 phase. Thus, we cannot, at present, determine when *FKS1* and *FKS2* promoter activity decreases back to its G1 levels.

A key question that still remains unanswered is whether and how Fks1 and Fks2 interact with each other to form functional GS, and it is very likely that the relative expression of the two genes under different conditions affects this process. The current paradigm, which is based on studies done in *S. cerevisiae* growing in culture, is that the Fks1 subunit of GS is predominantly expressed during unstressed growth, whereas Fks2 is induced by stress such as nutrient limitation and is required for specialized processes of mating and meiosis (both apparently absent in *C. glabrata*). However, unlike *S. cerevisiae*, *C. glabrata* is a commensal organism and an opportunistic pathogen adapted to growth under host-imposed stresses, which include nutrient limitation. Indeed, our RNA-seq mining results confirm that in the context of the host, Fks2 may be the more highly expressed GS subunit and may therefore play a more important role. This observation may also help explain why, in *C. glabrata*, the majority of echinocandin resistance-causing mutations in clinical strains are found in *FKS2* (8, 12, 15). Specifically, if GS is predominantly composed of the Fks2 subunit *in vivo*, then mutations in Fks2 hot-spot regions may have a greater effect on echinocandin resistance than mutations in the lesser-expressed Fks1 and may therefore be selected during treatment.

Altogether, our study contributes new insights into how the expression of the two GS subunits is regulated both *in vitro* and *in vivo* and may help explain the predominant involvement of mutations in *FKS2* in clinical echinocandin resistance.

## MATERIALS AND METHODS

### Yeast strains and media

The *C. glabrata* strains used in this study are described in Table S1. Cells were cultured in standard yeast extract-peptone-dextrose (YPD) medium (Thomas Scientific) or in synthetic complete (SC) medium containing glucose, yeast nitrogen base with ammonium sulfate, and amino acids (made as described [31]) at 37°C.

### Construction of transcriptional reporter plasmids

First, the YFP ORF on plasmid pYC55 (32) was replaced by the degGFP ORF from plasmid pNC1124 (20) using EcoRV and SacI cut sites. Next, 1 Kb of DNA sequence upstream of *FKS1* or *FKS2* ORFs was cloned into the EcoRV site of the resulting plasmid to generate p*FKS1-degGFP* and p*FKS2-degGFP*. The plasmids used in this study are listed in Table S2, and the primers used for the cloning are listed in Table S3.

### Time-lapse microscopy

Yeast cells harboring p*FKS1-degGFP* or p*FKS2-degGFP* were placed on pads containing SC medium with 1% SeaPlaque low-melting agarose (Lonza, Rockland, ME, USA), covered with a glass coverslip, and sealed using CoverGrip Coverslip Sealant (Biotube). The cells were imaged on a Nikon Eclipse Ti2 inverted microscope with a Hamamatsu ORCA-Flash4.0 camera using the 470 nm (FITC) filter at 60× magnification and 80 ms exposure time. Images of multiple fields were obtained every 20 min during a 3 h time period. The time-lapse images were analyzed using NIS-Elements software. First, each set of images was examined to identify cells that stayed in a single focal plane and underwent division during the imaging period. Next, for each cell of interest, all Z-stack planes containing the cell were merged using the maximal projection tool. Finally, the region of interest (ROI) tool was used to outline every selected cell individually, and its GFP signal intensity was determined by subtracting the background fluorescence signal from the internal cellular fluorescence signal using general automated image analysis.

### Construction of *C. glabrata fks1* and *fks2* point mutants

We selected two mutations in Fks1, S629P, and R631G, and two mutations in Fks2, S663P, and R665G. The specific codons underlying these mutations were adopted from previous studies (33, 34). To introduce each mutation, we used two overlapping ultramer primers in which the codon with the desired mutation was introduced. Of note, the PAM site was also mutated using silent codon changes to prevent CRISPR-Cas9 from cutting the amplicons. For each mutation, we carried out two PCRs using a forward primer with a reverse ultramer primer as well as the forward ultramer primer with the reverse primer (Table S3). Subsequently, these two PCR products were fused together, using short forward and reverse primers, purified, and checked using Sanger sequencing. Each fused PCR product was confirmed to carry two mutations: a non-synonymous mutation conferring echinocandin resistance and a silent mutation in the PAM site. Competent *C. glabrata* cells were prepared using the Frozen-EZ Yeast Transformation Kit (Zymo Research), and transformation followed an electroporation-based protocol described previously (35). The transformants were plated on YPD plates containing 0.125 µg/mL of micafungin and incubated for 1 week at 37°C. Positive colonies were subjected to PCR using diagnostic primers (Table S3), and the desired mutations were confirmed by Sanger sequencing.

### Construction of *fks1∆* and *fks2∆* mutants

*FKS1* and *FKS2* deletion mutants were generated in-house using a CRISPR-Cas9-targeted integration replacing the desired open reading frame (ORF) with a nourseothricin (NAT) resistance cassette by following a protocol described previously (35). Briefly, PCR products containing NAT-resistant gene and flanking regions complementary to *fks*1 and *fks*2 were generated using ultramer primers for Δ*fks*1 and Δ*fks2*, and regular primers for IGCg11, as in this case the complementary regions, together with the NAT-resistant gene, were directly amplified from the Δ*fks*1 strain previously generated. All primers are listed in Table S3. Electroporation-based transformation followed the previously described protocol (35). Transformants were selected on NAT-containing plates and validated by PCR amplification and sequencing of the targeted locus using external primers (Table S3). At least two independent transformants were generated and analyzed. All primers were ordered from Integrated DNA Technologies, and all Sanger sequencing of the above-described constructs was done by Genewiz.

### Construction of Halo-Tag Strains

For generating MVKCg6 and IGCg11 strains (Table S1), competent cells were prepared following a previously described method (36) with modifications. Briefly, cells were grown in YPD until OD_600_ = 1.8–2.5 to total 75 OD_600_ and then harvested and resuspended in conditioning buffer (100 mM LiAc, 10 mM Tris-HCl, 1 mM EDTA) to 3.8 OD_600_. Next, cells were incubated at 37°C with shaking for 1 h and another 30 min after the addition of DTT to 10 mM. Cells were washed twice with Milli-Q water and once in 1M sorbitol and re-suspended in 200 µL of 1M sorbitol. CRISPR-Cas9 was performed by electroporation in 2 mm gap cuvettes at 1.8 kV, using a mixture consisting of 40 µL of cell slurry, 0.8-1.2 µg of EDTA-free DNA fragment in 5 µL and 6.6 µL of assembled Cas9-RNA complex (3.3 µL of mixture of 4 µM tracrRNA and 4 µM crRNA, and 3 µL of 4 µM of Alt-R HiFi Cas9 Nuclease (Integrated DNA Technologies); 1 mL of 1M sorbitol was added immediately after electroporation. After 5 min at RT, cells were spun down at 2000 g 5 min and re-suspended in 2 mL of YPD. Cells were allowed to recover for 2.5 h at 30°C before plating on selective media. To construct strain MVKCg6, CBS138 strain was used as a host in which the flanking cassette (hygromycin (HYG) selective marker, 1,000 bp of FKS2 promoter, N-terminal yeast codon-optimized HaloTag (derived from Addgene plasmid 177102 YIplac211-ENT5-HaloTag) and 4× alanine linker) was introduced upstream of the first codon of the *FKS2* gene through homologous recombination. To construct strain IGCg11, MVKCg6 was used as the host strain in which *fks*1 was replaced by a NAT resistance cassette.

### RNA extraction and quantitative real-time reverse-transcription PCR (qRT-PCR)

Overnight cultures of *C. glabrata* were adjusted to 1 OD_600_ in fresh YPD containing FK506 (1, 2, 4, and 8 µg/mL) or caspofungin (0.005, 0.01, or 0.02 µg/mL) and incubated for 1 h. After that, cultures were harvested using TRIzol LS Reagent (Invitrogen), and RNA was extracted using the RNeasy Mini Kit (Qiagen Science), following the manufacturer’s instructions. Then, RNA was treated with Turbo DNase (Invitrogen) according to the manufacturer’s recommendations. RNA samples were stored at −80°C. The transcript levels of FKS1, FKS2, PTP3, and RCN2 were measured by RT-PCR using one-step TB Green PrimeScript RT-PCR kit II (TaKaRa). Reactions were run on an AriaMx qPCR System (Agilent Technologies) containing 10 ng of the RNA sample, 0.4 µM of each primer (Table S3), 12.5 µL of 2× one-step TB Green RT-PCR buffer, and 1 µL of PrimeScript 1 step enzyme mix 2 in a volume of 25 µL. Thermal cycling conditions were 42°C for 5 min for the reverse transcription and PCR cycling with initial denaturation at 95°C for 10 s, followed by 40 cycles of denaturation at 95°C for 5 s and annealing and elongation at 60°C for 30 s; a post-PCR melting curve analysis with 95°C for 5 s; 60°C for 1 min; and then increasing to 95°C. Each experiment was carried out with at least three biological replicates, and at least two technical replicates of each biological replicate were also performed. Negative controls were included in each run. The UBC13 gene was used as the reference gene to normalize the data (37). Comparative expression analyses were performed using the threshold cycle (2^-ΔCt^ method) (38). The fold changes were determined from the normalized expression of the samples relative to the housekeeping gene.

### Generating *FKS2* sashimi plot

To produce the sashimi plots, STAR (v2.7.10a) .bam output was indexed, quantified for coverage at nucleotide level, and averaged to obtain the median value using “pysam.AlignmentFile.count_coverage” function and then smoothed over 50 bp sliding window using pysam (v0.19.1) and Matplotlib (v3.5.1).

### Flow cytometry analysis of GFP and JF549 fluorescence

Overnight cultures of *C. glabrata* carrying the *pFKS-degGFP* plasmids were diluted in fresh YPD containing the appropriate antibiotic (i.e., nourseothricin [Gold Biotechnology] or hygromycin [Gold Biotechnology]) and incubated at 37°C for 3 hours. Next, culture OD_600_ was adjusted to 1 in liquid YPD containing, when required, FK506 (1, 2, 4, and 8 µg/mL) and incubated for 1 h. The cells were then washed twice with phosphate-buffered saline (PBS) and stained with 10 mg/mL propidium iodide (PI) to gate out dead cells. For HT-Fks2 quantification, the same protocol was followed with a few modifications. After the 3 h of incubation at 37°C, cells were adjusted to an OD_600_ of 0.2 in YPD and incubated with 200 µM Janelia Fluor HaloTag Ligand 549 (JF549, Promega) for 30 min at 37°C. Next, cells were washed with PBS and analyzed by flow cytometry. PI staining was not used in this instance because PI and JF549 staining cannot be fully distinguished by flow cytometry. Flow cytometry was performed using a LSRFortessa cytometer (BD Bioscience). Data were analyzed using FlowJo_v10.8.1.

### Microscopy analysis of HT-Fks2 localization

Logarithmically growing cells expressing HT-Fks2 from its endogenous chromosomal locus (see construction details above) were incubated with 500 µM Janelia Fluor HaloTag Ligand 549 (JF549, Promega) for 20 min at 37°C, washed with PBS, and imaged using the TRITC filter (100 ms exposure) and bright-field (25 ms exposure) of a Nikon Eclipse Ti2 inverted microscope with a Hamamatsu ORCA-Flash4.0 camera.

### Extracting *FKS1* and *FKS2* RNA-seq read counts

RNA-seq data were aligned to the *C. glabrata* CBS138 (GCA000002545v2) reference genome; genome reference as well as the coordinates in .gtf format for FKS1 (CAGL0G01034g) and FKS2 (CAGL0K04037g) were obtained from the Candida Genome Database (39). Reads were aligned using STAR (v2.7.10a) under parameters “--outSAMtype BAM SortedByCoordinate—quantMode GeneCounts.” read counts per gene were extracted from ReadsPerGene.out.tab output.

### Statistical analysis

All shown results represent an average of three or more independent experiments (biological replicates). Error bars represent the standard deviation. All data were analyzed using GraphPad Prism 8 software. Statistical analyses were performed using an unpaired *t*-test, ANOVA, or the Kruskal-Wallis test, depending on the number of groups being compared and the normality of distribution. Normality was determined using the Shapiro-Wilk test. *P* values of ≤ 0.05 were considered statistically significant.

## SUPPLEMENTAL MATERIAL

Fig. S1 aac.00500-25-s0001.tif Depiction of the flow cytometry scheme used to measure the fluorescence intensity of pFKS1/2-degGFP reporters in culture.

Fig. S2 aac.00500-25-s0002.tif qRT-PCR showed no significant effects of *fks2*∆ or FK506 on FKS1 expression.

Fig. S3 aac.00500-25-s0003.tif The HaloTag did not interfere with Fks2 function, as reflected by HT-Fks2-S663P retaining the echinocandin-resistant phenotype of Fks2-S663P.

Supplemental material aac.00500-25-s0004.docx Legends for Fig. S1 to S3.

Supplemental tables aac.00500-25-s0005.xlsx Tables S1 to S3.

ASM does not own the copyrights to Supplemental Material that may be linked to, or accessed through, an article. The authors have granted ASM a non-exclusive, world-wide license to publish the Supplemental Material files. Please contact the corresponding author directly for reuse.

## References

[1] Cornely OA, Sprute R, Bassetti M, Chen SC-A, Groll AH, Kurzai O, Lass-Flörl C, Ostrosky-Zeichner L, Rautemaa-Richardson R, Revathi G, et al.. 2025. Global guideline for the diagnosis and management of candidiasis: an initiative of the ECMM in cooperation with ISHAM and ASM. Lancet Infect Dis 25:e280–e293. doi:10.1016/S1473-3099(24)00749-7 39956121

[2] Alexander BD, Johnson MD, Pfeiffer CD, Jiménez-Ortigosa C, Catania J, Booker R, Castanheira M, Messer SA, Perlin DS, Pfaller MA. 2013. Increasing echinocandin resistance in Candida glabrata: clinical failure correlates with presence of FKS mutations and elevated minimum inhibitory concentrations. Clin Infect Dis 56:1724–1732. doi:10.1093/cid/cit136 23487382 PMC3658363

[3] Barber AE, Weber M, Kaerger K, Linde J, Gölz H, Duerschmied D, Markert A, Guthke R, Walther G, Kurzai O. 2019. Comparative genomics of serial Candida glabrata isolates and the rapid acquisition of echinocandin resistance during therapy. Antimicrob Agents Chemother 63:e01628-18. doi:10.1128/AAC.01628-18 30478162 PMC6355595

[4] Healey KR, Perlin DS. 2018. Fungal resistance to echinocandins and the MDR phenomenon in Candida glabrata. J Fungi (Basel) 4:105. doi:10.3390/jof4030105 30200517 PMC6162769

[5] Lewis JS, Wiederhold NP, Wickes BL, Patterson TF, Jorgensen JH. 2013. Rapid emergence of echinocandin resistance in Candida glabrata resulting in clinical and microbiologic failure. Antimicrob Agents Chemother 57:4559–4561. doi:10.1128/AAC.01144-13 23817368 PMC3754289

[6] Pfaller MA, Castanheira M, Lockhart SR, Ahlquist AM, Messer SA, Jones RN. 2012. Frequency of decreased susceptibility and resistance to echinocandins among fluconazole-resistant bloodstream isolates of Candida glabrata. J Clin Microbiol 50:1199–1203. doi:10.1128/JCM.06112-11 22278842 PMC3318516

[7] Sasso M, Roger C, Lachaud L. 2017. Rapid emergence of FKS mutations in Candida glabrata isolates in a peritoneal candidiasis. Med Mycol Case Rep 16:28–30. doi:10.1016/j.mmcr.2017.04.004 28491490 PMC5413194

[8] Aldejohann AM, Herz M, Martin R, Walther G, Kurzai O. 2021. Emergence of resistant Candida glabrata in Germany. JAC Antimicrob Resist 3:dlab122. doi:10.1093/jacamr/dlab122 34377983 PMC8346698

[9] Khalifa HO, Arai T, Majima H, Watanabe A, Kamei K. 2020. Genetic basis of azole and echinocandin resistance in clinical Candida glabrata in Japan. Antimicrob Agents Chemother 64:e00783-20. doi:10.1128/AAC.00783-20 32571826 PMC7449192

[10] Vallabhaneni S, Cleveland AA, Farley MM, Harrison LH, Schaffner W, Beldavs ZG, Derado G, Pham CD, Lockhart SR, Smith RM. 2015. Epidemiology and risk factors for echinocandin nonsusceptible Candida glabrata bloodstream infections: data from a large multisite population-based candidemia surveillance program, 2008-2014. Open Forum Infect Dis 2:ofv163. doi:10.1093/ofid/ofv163 26677456 PMC4677623

[11] Garcia-Effron G, Lee S, Park S, Cleary JD, Perlin DS. 2009. Effect of Candida glabrata FKS1 and FKS2 mutations on echinocandin sensitivity and kinetics of 1,3-beta-D-glucan synthase: implication for the existing susceptibility breakpoint. Antimicrob Agents Chemother 53:3690–3699. doi:10.1128/AAC.00443-09 19546367 PMC2737881

[12] Pham CD, Iqbal N, Bolden CB, Kuykendall RJ, Harrison LH, Farley MM, Schaffner W, Beldavs ZG, Chiller TM, Park BJ, Cleveland AA, Lockhart SR. 2014. Role of FKS mutations in Candida glabrata: MIC values, echinocandin resistance, and multidrug resistance. Antimicrob Agents Chemother 58:4690–4696. doi:10.1128/AAC.03255-14 24890592 PMC4136002

[13] Katiyar SK, Alastruey-Izquierdo A, Healey KR, Johnson ME, Perlin DS, Edlind TD. 2012. Fks1 and Fks2 are functionally redundant but differentially regulated in Candida glabrata: implications for echinocandin resistance. Antimicrob Agents Chemother 56:6304–6309. doi:10.1128/AAC.00813-12 23027185 PMC3497156

[14] Mazur P, Morin N, Baginsky W, el-Sherbeini M, Clemas JA, Nielsen JB, Foor F. 1995. Differential expression and function of two homologous subunits of yeast 1,3-beta-D-glucan synthase. Mol Cell Biol 15:5671–5681. doi:10.1128/MCB.15.10.5671 7565718 PMC230817

[15] Misas E, Seagle E, Jenkins EN, Rajeev M, Hurst S, Nunnally NS, Bentz ML, Lyman MM, Berkow E, Harrison LH, Schaffner W, Markus TM, Pierce R, Farley MM, Chow NA, Lockhart SR, Litvintseva AP. 2024. Genomic description of acquired fluconazole- and echinocandin-resistance in patients with serial Candida glabrata isolates. J Clin Microbiol 62:e0114023. doi:10.1128/jcm.01140-23 38265207 PMC10865870

[16] Zhao C, Jung US, Garrett-Engele P, Roe T, Cyert MS, Levin DE. 1998. Temperature-induced expression of yeast FKS2 is under the dual control of protein kinase C and calcineurin. Mol Cell Biol 18:1013–1022. doi:10.1128/MCB.18.2.1013 9447998 PMC108813

[17] Healey KR, Paderu P, Hou X, Jimenez Ortigosa C, Bagley N, Patel B, Zhao Y, Perlin DS. 2020. Differential regulation of echinocandin targets Fks1 and Fks2 in Candida glabrata by the post-transcriptional regulator Ssd1. J Fungi (Basel) 6:143. doi:10.3390/jof6030143 32825653 PMC7558938

[18] Wang Q, Li Y, Cai X, Li R, Zheng B, Yang E, Liang T, Yang X, Wan Z, Liu W. 2021. Two sequential clinical isolates of Candida glabrata with multidrug-resistance to posaconazole and echinocandins. Antibiotics (Basel) 10:1217. doi:10.3390/antibiotics10101217 34680798 PMC8532709

[19] Niimi K, Woods MA, Maki K, Nakayama H, Hatakenaka K, Chibana H, Ikeda F, Ueno K, Niimi M, Cannon RD, Monk BC. 2012. Reconstitution of high-level micafungin resistance detected in a clinical isolate of Candida glabrata identifies functional homozygosity in glucan synthase gene expression. J Antimicrob Chemother 67:1666–1676. doi:10.1093/jac/dks112 22514266

[20] Houser JR, Ford E, Chatterjea SM, Maleri S, Elston TC, Errede B. 2012. An improved short‐lived fluorescent protein transcriptional reporter for Saccharomyces cerevisiae. Yeast 29:519–530. doi:10.1002/yea.2932 23172645 PMC3521066

[21] Turner JJ, Ewald JC, Skotheim JM. 2012. Cell size control in yeast. Curr Biol 22:R350–9. doi:10.1016/j.cub.2012.02.041 22575477 PMC3350643

[22] Zimbeck AJ, Iqbal N, Ahlquist AM, Farley MM, Harrison LH, Chiller T, Lockhart SR. 2010. FKS mutations and elevated echinocandin MIC values among Candida glabrata isolates from U.S. population-based surveillance. Antimicrob Agents Chemother 54:5042–5047. doi:10.1128/AAC.00836-10 20837754 PMC2981257

[23] Garcia-Effron G, Katiyar SK, Park S, Edlind TD, Perlin DS. 2008. A naturally occurring proline-to-alanine amino acid change in Fks1p in Candida parapsilosis, Candida orthopsilosis, and Candida metapsilosis accounts for reduced echinocandin susceptibility. Antimicrob Agents Chemother 52:2305–2312. doi:10.1128/AAC.00262-08 18443110 PMC2443908

[24] Los GV, Encell LP, McDougall MG, Hartzell DD, Karassina N, Zimprich C, Wood MG, Learish R, Ohana RF, Urh M, Simpson D, Mendez J, Zimmerman K, Otto P, Vidugiris G, Zhu J, Darzins A, Klaubert DH, Bulleit RF, Wood KV. 2008. HaloTag: a novel protein labeling technology for cell imaging and protein analysis. ACS Chem Biol 3:373–382. doi:10.1021/cb800025k 18533659

[25] Williams RB, Lorenz MC. 2020. Multiple alternative carbon pathways combine to promote Candida albicans stress resistance, immune interactions, and virulence. MBio 11:e03070-19. doi:10.1128/mBio.03070-19 31937647 PMC6960290

[26] Arastehfar A, Daneshnia F, Hovhannisyan H, Fuentes D, Cabrera N, Quinteros C, Ilkit M, Ünal N, Hilmioğlu-Polat S, Jabeen K, Zaka S, Desai JV, Lass-Flörl C, Shor E, Gabaldon T, Perlin DS. 2023. Overlooked Candida glabrata petites are echinocandin tolerant, induce host inflammatory responses, and display poor in vivo fitness. MBio 14:e0118023. doi:10.1128/mbio.01180-23 37772846 PMC10653939

[27] Mo X, Yu X, Cui H, Xiong K, Yang S, Su C. 2024. In vivo RNA sequencing reveals a crucial role of Fus3-Kss1 MAPK pathway in Candida glabrata pathogenicity. mSphere 9:e0071524. doi:10.1128/msphere.00715-24 39475321 PMC11580445

[28] Kalem MC, Subbiah H, Leipheimer J, Glazier VE, Panepinto JC. 2021. Puf4 mediates post-transcriptional regulation of cell wall biosynthesis and caspofungin resistance in Cryptococcus neoformans. MBio 12:e03225-20. doi:10.1128/mBio.03225-20 33436441 PMC7844544

[29] Arastehfar A, Daneshnia F, Cabrera N, Penalva-Lopez S, Sarathy J, Zimmerman M, Shor E, Perlin DS. 2023. Macrophage internalization creates a multidrug-tolerant fungal persister reservoir and facilitates the emergence of drug resistance. Nat Commun 14:1183. doi:10.1038/s41467-023-36882-6 36864040 PMC9981703

[30] Howard SJ, Livermore J, Sharp A, Goodwin J, Gregson L, Alastruey-Izquierdo A, Perlin DS, Warn PA, Hope WW. 2011. Pharmacodynamics of echinocandins against Candida glabrata: requirement for dosage escalation to achieve maximal antifungal activity in neutropenic hosts. Antimicrob Agents Chemother 55:4880–4887. doi:10.1128/AAC.00621-11 21807969 PMC3186993

[31] Synthetic defined medium with amino acids. 2015. Cold Spring Harbor Laboratory Press. Available from: https://cshprotocols.cshlp.org/content/2015/2/pdb.rec085639.full

[32] Yáñez-Carrillo P, Orta-Zavalza E, Gutiérrez-Escobedo G, Patrón-Soberano A, De Las Peñas A, Castaño I. 2015. Expression vectors for C-terminal fusions with fluorescent proteins and epitope tags in Candida glabrata. Fungal Genet Biol 80:43–52. doi:10.1016/j.fgb.2015.04.020 25986172

[33] Arastehfar A, Daneshnia F, Zomorodian K, Najafzadeh MJ, Khodavaisy S, Zarrinfar H, Hagen F, Zare Shahrabadi Z, Lackner M, Mirhendi H, Salehi M, Roudbary M, Pan W, Kostrzewa M, Boekhout T. 2019. Low level of antifungal resistance in Iranian isolates of Candida glabrata recovered from blood samples in a multicenter study from 2015 to 2018 and potential prognostic values of genotyping and sequencing of PDR1. Antimicrob Agents Chemother 63:e02503-18. doi:10.1128/AAC.02503-18 30936110 PMC6591624

[34] Arastehfar A, Daneshnia F, Salehi M, Yaşar M, Hoşbul T, Ilkit M, Pan W, Hagen F, Arslan N, Türk-Dağı H, Hilmioğlu-Polat S, Perlin DS, Lass-Flörl C. 2020. Low level of antifungal resistance of Candida glabrata blood isolates in Turkey: fluconazole minimum inhibitory concentration and FKS mutations can predict therapeutic failure. Mycoses 63:911–920. doi:10.1111/myc.13104 32413170 PMC7497236

[35] Shor E, Schuyler J, Perlin DS. 2019. A novel, drug resistance-independent, fluorescence-based approach to measure mutation rates in microbial pathogens. MBio 10:e00120-19. doi:10.1128/mBio.00120-19 30808701 PMC6391916

[36] Grahl N, Demers EG, Crocker AW, Hogan DA. 2017. Use of RNA-protein complexes for genome editing in non-albicans Candida species. mSphere 2:e00218-17. doi:10.1128/mSphere.00218-17 28657070 PMC5480035

[37] Li QQ, Skinner J, Bennett JE. 2012. Evaluation of reference genes for real-time quantitative PCR studies in Candida glabrata following azole treatment. BMC Mol Biol 13:22. doi:10.1186/1471-2199-13-22 22747760 PMC3482582

[38] Livak KJ, Schmittgen TD. 2001. Analysis of relative gene expression data using real-time quantitative PCR and the 2(-Delta Delta C(T)) Method. Methods 25:402–408. doi:10.1006/meth.2001.1262 11846609

[39] Skrzypek MS, Binkley J, Binkley G, Miyasato SR, Simison M, Sherlock G. 2017. The Candida Genome Database (CGD): incorporation of Assembly 22, systematic identifiers and visualization of high throughput sequencing data. Nucleic Acids Res 45:D592–D596. doi:10.1093/nar/gkw924 27738138 PMC5210628

