# Supplementary figures and images for: Expression of 1,3-β-glucan synthase subunits in Candida glabrata is regulated by the cell cycle and growth conditions and at both transcriptional and post-transcriptional levels
Source: Antimicrob Agents Chemother. 2025 Jun 17;69(8):e00500-25. doi: 10.1128/aac.00500-25 (PMC12326982; doi:10.1128/aac.00500-25)

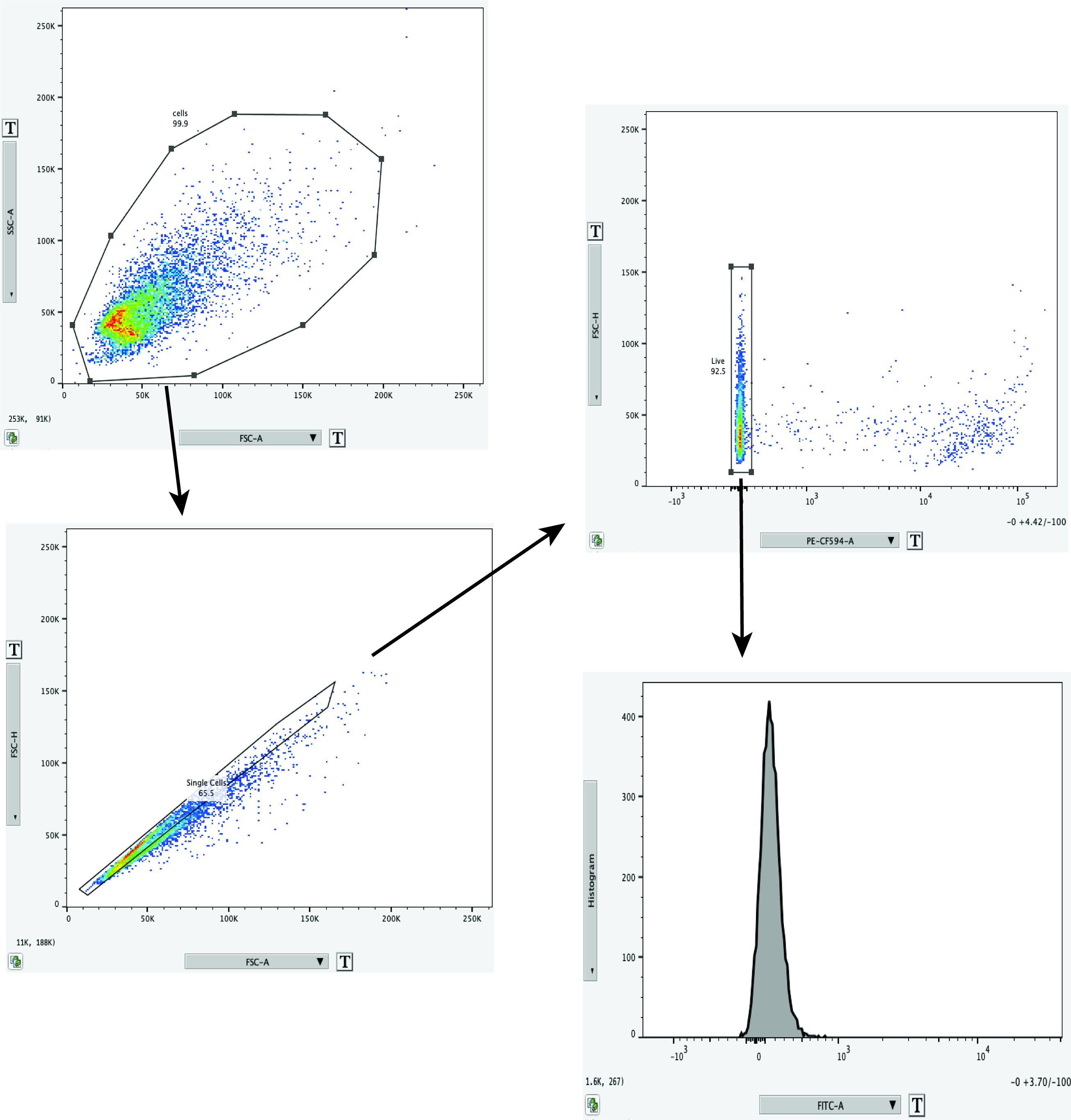

Supplement: Fig. S1 — Depiction of the flow cytometry scheme used to measure the fluorescence intensity of pFKS1/2-degGFP reporters in culture. [file aac.00500-25-s0001.tif]

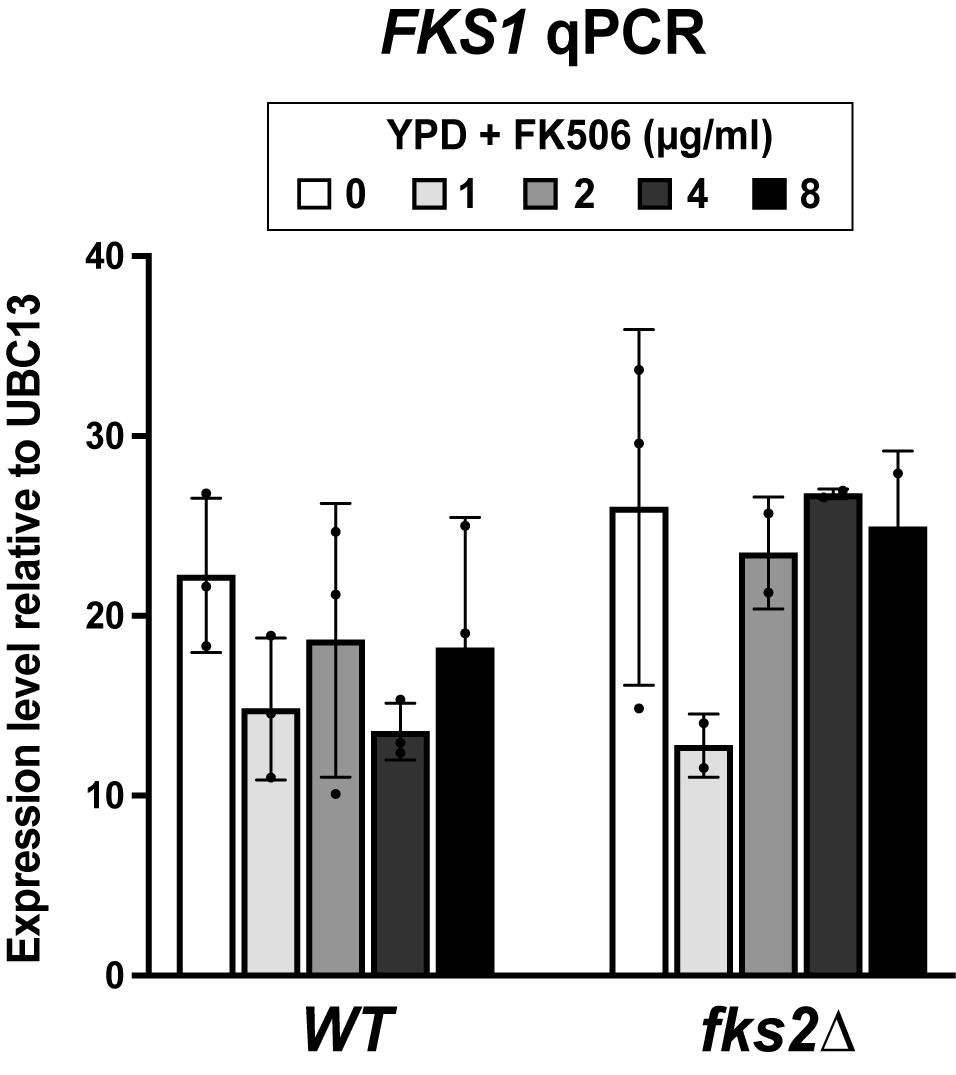

Supplement: Fig. S2 — qRT-PCR showed no significant effects of fks2∆ or FK506 on FKS1 expression. [file aac.00500-25-s0002.tif]

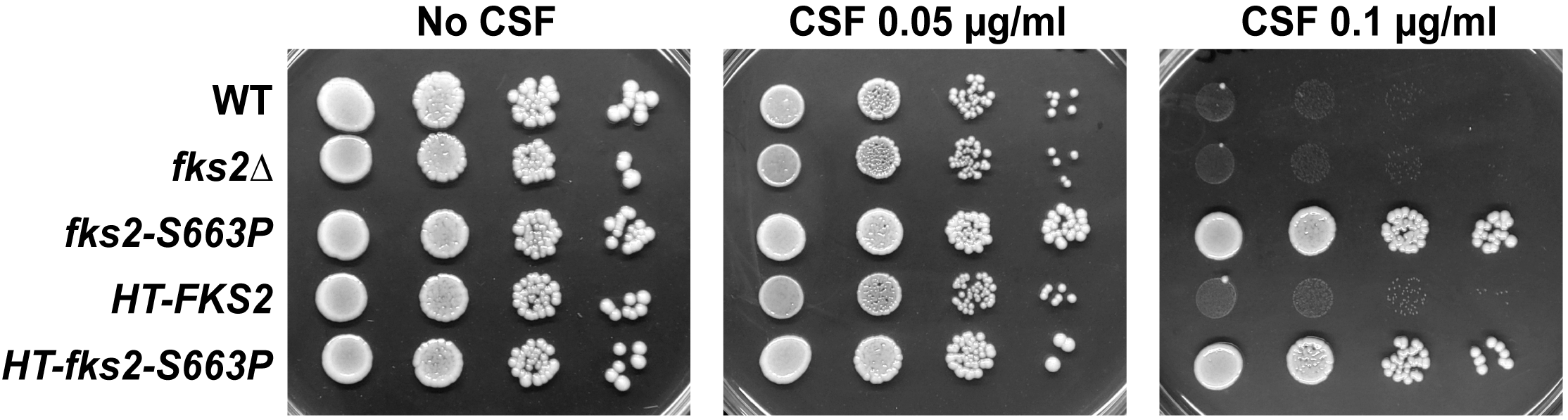

Supplement: Fig. S3 — The HaloTag did not interfere with Fks2 function, as reflected by HT-Fks2-S663P retaining the echinocandin-resistant phenotype of Fks2-S663P. [file aac.00500-25-s0003.tif]
