# Supplementary material for: Expression of 1,3-β-glucan synthase subunits in Candida glabrata is regulated by the cell cycle and growth conditions and at both transcriptional and post-transcriptional levels
Source: Antimicrob Agents Chemother. 2025 Jun 17;69(8):e00500-25. doi: 10.1128/aac.00500-25 (PMC12326982; doi:10.1128/aac.00500-25)
Supplement: Supplemental material — Legends for Fig. S1 to S3. [file aac.00500-25-s0004.docx]

**Figure S1. Depiction of the flow cytometry scheme used to measure the fluorescence intensity of p*FKS1/2-degGFP* reporters in culture.** Doublets were excluded by plotting FSC-H vs FSC-A and propidium iodide staining was used to exclude dead (dye-positive) cells (PE-CF594 signal).

**Figure S2. qRT-PCR showed no significant effects of *fks2∆* or FK506 on *FKS1* expression.**

**Figure S3. The HaloTag (HT) did not interfere with Fks2 function, as reflected by HT-Fks2-S663P retaining the echinocandin-resistant phenotype of Fks2-S663P.** Serial dilutions of the indicated strains were spotted on the plates, cultured at 37°C, and photographed after two days.
